# The primary visual cortex of Cetartiodactyls: organization, cytoarchitectonics and comparison with perissodactyls and primates

**DOI:** 10.1007/s00429-021-02392-8

**Published:** 2021-10-03

**Authors:** Jean-Marie Graïc, Antonella Peruffo, Livio Corain, Livio Finos, Enrico Grisan, Bruno Cozzi

**Affiliations:** 1 Department of Comparative Biomedicine and Food Science, University of Padova, Viale dell’Università 16 Legnaro, 35020 Padova, PD Italy; 2 Department of Management and Engineering, University of Padova, Padova, Italy; 3 Department of Developmental Psychology and Socialization, University of Padova, Padova, Italy; 4 Department of Information Engineering, University of Padova, Padova, Italy; 5 School of Engineering, London South Bank University, London, SE1 0AA UK

**Keywords:** Visual cortex, Comparative neuroanatomy, Cetartiodactyls, Lateralization, Lamination, Cytoarchitecture

## Abstract

Cetartiodactyls include terrestrial and marine species, all generally endowed with a comparatively lateral position of their eyes and a relatively limited binocular field of vision. To this day, our understanding of the visual system in mammals beyond the few studied animal models remains limited. In the present study, we examined the primary visual cortex of Cetartiodactyls that live on land (sheep, Père David deer, giraffe); in the sea (bottlenose dolphin, Risso’s dolphin, long-finned pilot whale, Cuvier’s beaked whale, sperm whale and fin whale); or in an amphibious environment (hippopotamus). We also sampled and studied the visual cortex of the horse (a closely related perissodactyl) and two primates (chimpanzee and pig-tailed macaque) for comparison. Our histochemical and immunohistochemical results indicate that the visual cortex of Cetartiodactyls is characterized by a peculiar organization, structure, and complexity of the cortical column. We noted a general lesser lamination compared to simians, with diminished density, and an apparent simplification of the intra- and extra-columnar connections. The presence and distribution of calcium-binding proteins indicated a notable absence of parvalbumin in water species and a strong reduction of layer 4, usually enlarged in the striated cortex, seemingly replaced by a more diffuse distribution in neighboring layers. Consequently, thalamo-cortical inputs are apparently directed to the higher layers of the column. Computer analyses and statistical evaluation of the data confirmed the results and indicated a substantial correlation between eye placement and cortical structure, with a markedly segregated pattern in cetaceans compared to other mammals. Furthermore, cetacean species showed several types of cortical lamination which may reflect differences in function, possibly related to depth of foraging and consequent progressive disappearance of light, and increased importance of echolocation.

## Introduction

Vision is a key asset for most mammals, depending on their habitat and evolution. From the hedgehog, a model for the ancestral mammalian brain, to primates, there has been considerable nonlinear phylogenetic specialization of the visual system, depending on prey *vs.* predatory status, night *vs.* day activity, terrestrial *vs.* aquatic life and so forth. Although the mammalian visual sensory system has been extensively investigated, studies on species variations are still scarce. Most lines of research focused on primates (Livingstone and Hubel 1984, 1988; Lund 1987; Lund et al. 1988, 1991, 1997; Barton 1998; Van Essen et al. 1992; Goebel et al. 2012), cats (Rosenquist 1985) and rodents (for references see Pearlman 1985), although earlier works also included other mammalian orders (van Sluyters and Stewart 1974; Clarke et al. 1979a, b; Duke-Elder 1961; Hebel 1976; Karamanlidis 1979).

Different taxa reveal a different organization of their visual system. Primates are characterized in part by their pronounced binocularity, an understandable evolution for mammals with a prehensile hand (Szalay 1975). Carnivores possess a certain degree of binocularity attributed to their predatory activities (Hughes 1977). Terrestrial Cetartiodactyls are herbivore species that developed a phylogenetic adaptation to extremely contrasting habitats, from arid land to water, with consequent various degrees of differential evolution of their visual system and other senses. The horse is a perissodactyl that shares several adaptive features with most terrestrial Cetartiodactyls, including the general morphology of the visual system (Harman et al. 1999; Timney and Kiel 1999; Kendrick et al. 2001; Hall et al. 2003; Knolle et al. 2017). All Cetartiodactyls (including sea-living cetaceans) and perissodactyls developed a broad field of view mostly relying on monocular signal (table, Johnson 1901; Duke-Elder 1958), although their vision has been deemed fairly good (Piggins et al. 1996, Jacobs et al. 1998; Coimbra et al. 2013, 2017). However, most of these species are also endowed with a binocular field (Duke-Elder 1958; Hughes and Whitteridge 1973; Clarke et al. 1979b; Timney and Kiel 1999); and pioneer experiments found binocular neurons in the visual cortex of the sheep and goat (Clarke and Whitteridge 1976; Clarke et al. 1976, 1979a,b). Notwithstanding these latter studies, knowledge on the visual system of ungulates remains relatively limited. The visual system of cetaceans, i.e., marine Cetartiodactyla, drew some attention (Cuvier 1836; Kellogg 1938; Rochon-Duvigneaud 1939; Duke-Elder 1958; Walls 1963; Dral 1972, 1975; Herman et al. 1975), because the eyes are placed almost exactly opposite to each other and appear to be able to move independently (Dawson et al. 1981). Binocular vision in cetaceans is therefore necessarily extremely limited (Dawson et al. 1981; Garey et al. 1985; Mobley and Helweg 1990; von Fersen et al. 2000; Kilian et al. 2000), but remains possible, given the examples of binocular vision demonstrated in other lateral-eyes species (Holden and Low 1989). Independence of the eyes implies that we should expect complete decussation of the optic nerves, which has been observed in very few specimens, albeit with conflicting reports (Ridgeway 1990 citing Hatschek 1903 and Jacobs et al. 1964, 1975; Korneliussen, personal communication in Morgane and Jacobs 1972).

The mammalian primary visual cortex (V1) corresponds to Brodmann’s area 17 and is the target of the geniculo-calcarine tract. Six distinct layers are commonly recognized in V1, according to Brodmann’s original description (Brodmann 1909), including a further subdivision of layer 4 (L4) in 3 sublayers. The V1 of primates is also called “striate cortex” because of the presence of the line of Gennari (Gennari 1782), visible macroscopically as a stripe (Ström and Ekesten 2016). The line is formed by myelinated axons that originate in the lateral geniculate nucleus (LGN) and synapse in the inner granular layer (L4) of the cortical gray matter, in sublayers 4Cα for magnocellular (M) projections and 4A and 4Cβ for parvocellular (P) projections (Fitzpartrick et al. 1985). This basic organization has since been thoroughly updated to include koniocellular (K) projections (Dacey and Lee 1994; Sincich and Horton 2005). Some other inputs to V1 are also found in L6, with both types of cells, but in a separate manner (Fitzpatrick and Einstein 1989; Lund and Boothe 1975).

The boundaries, however, of the striate and peristriate cortex have been extensively discussed since its original mapping (Brodmann 1909) and remained contradictory, up until the development of additional staining techniques (Braak 1977; Rosa and Krubitzer 1999; Amunts et al. 2000). The organization of V1 is the hallmark of the sensory cortex in primates, also called koniocortex, and is characterized by the presence of intense granularity in L2 and L4 (outer and inner granular layers). The lack of a classic koniocortex, as described in man, was noted by Sanides and Hoffmann (1969) in the cat, in which granules are substituted by larger cells. In fact, according to Brodmann (Brodmann 1909) area 17 concerns primates, and although the 6 layers are constant in all mammals, L4 has undergone such vast alterations across species that it is often overlooked, including in ungulates. As a matter of fact, the cytoarchitecture of V1 in ungulates has received little attention aside from mostly the sheep (Rose 1942; Karamanlidis 1979).

Although V1 is only a part of the complex visual apparatus which can take various forms (Duke Elder 1958), it is the main cortical stage for inputs from the LGN in mammals for the vast majority of the transmitted signal (Snicich and Horton 2005). V1 has recently been found to be the primary zone of activity for the early reaction to frightful stimuli, together with the pulvinar (Koizumi et al. 2019), which almost all animals experience. This indication suggests further study of the organization of the visual cortex in mammalian prey species, which potentially favor width of the visual field instead of binocular potential. For this reason, here we focused our attention on the V1 of several Cetartiodactyls, including the terrestrial giraffe (*Giraffa camelopardalis*), Pere Davide’s deer (*Elaphurus davidianus*), and sheep (*Ovis aries*), the semi-aquatic hippopotamus (*Hippopotamus amphibius*), and the marine bottlenose dolphin (*Tursiops truncatus*), Risso’s dolphin (*Grampus griseus*), long-finned pilot whale (*Globicephala melas*), Cuvier’s beaked whale (*Ziphius cavirostris*) sperm whale (*Physeter macrocephalus*) and fin whale (*Balaenoptera physalus*). Their visual systems share a typical feature, the presence of the visual streak (Hughes and Whitteridge 1973; Hughes 1977; Shinozaki et al. 2010; Coimbra et al. 2013, 2017), although its structure is somewhat different in cetaceans (Mass and Supin 2007). The temporal region of the retina of Cetartiodactyls is thought to facilitate frontal vision (Collin 1999; Hughes, 1977). However, the visual streak of terrestrial ungulates is functional to a panoramic horizontal view (Hughes 1977), while in cetaceans it specialized into two high resolution points in the retina, marked by higher ganglion cell density (Dawson and Perez 1973; Dral 1975; Herman et al. 1975; Dawson et al. 1982; Mass and Supin 2007). In the present study we also considered the horse, the typical representative of the perissodactyls, because its original environment, dietary habits and especially the position of the eyes, are similar to those of terrestrial Cetartiodactyls and a comparison of the organization of the respective visual systems may yield insights on the development of potentially similar phylogenetic adaptations. As a general reference we also examined the visual cortices of two primates, the chimpanzee, and the pig-tailed macaque.

## Materials and methods

### Animal tissues

The specimens were collected at the Department of Comparative Biomedicine and Food Science (BCA) of the University of Padova. The brains were fixed by immersion in 4% phosphate buffered formalin immediately after extraction. For large-brained specimens, the cerebrum was cut in thick slices to allow a faster fixation. The list of specimens used can be seen in Table 1.

**Table 1:** Sampling data, references on sampling topography and information on the visual field of each examined species (CC: conservation code, used in stranding protocols, Ijsseldijk et al. 2019)

Species		*N*	Post-mortem interval	Sampling Reference	Monocular field; binocular; reference	Cortical thickness ± s.d. (mm)
Primates						
*Macaca nemestrina*	Pig-tailed macaque	1	Immediate, perfused animal	Peters (1994)		1.657 ± 0.16
*Pan troglodytes*	Chimpanzee	2	6 h	Bailey et al. (1950); Allman and McGuinness (1988)		2.108 ± 0.29
Perissodactyls						
*Equus caballus*	Horse	2	6–12 h	Ström and Ekesten (2016)	320; 80; Harman et al. (1999) 65°; Duke-Elder (1958); Timney and Keil (1999)	2.041 ± 0.49
Terrestrial Cetartiodactyls						
*Ovis aries*	Sheep	3	2–4 h	Rose (1942); Clarke and Whitteridge (1976)	< 360; 60; Lindsay Johnson (1901)	1.488 ± 0.21
*Elaphurus davidianus*	Père David’s deer	2	6–12 h	Clarke and Whitteridge (1976)	50° of optical divergence; Lindsay Johnson 1901	1.947 ± 0.13
*Giraffa camelopardalis*	Giraffe	2	6–12 h	Jacobs et al. (2014)	75° of optical divergence; 60° overlap Lindsay Johnson (1901); Mitchell et al. (2013)	2.637 ± 0.31
Semi-aquatic Cetartiodactyls						
*Hippopotamus amphibius*	Hippopotamus	2	6–12 h	Butti et al. (2014a)	60° of optical divergence; Lindsay Johnson (1901)	2.205 ± 0.59
Marine Cetartiodactyls						
Toothed dolphins and whales (Odontocetes)						
*Tursiops truncatus*	Bottlenose dolphin	2	< 24 h from stranding (CC 1–2)	Sokolov et al. (1972); Kesarev et al. (1977); Ladygina et al. (1978)	20–30° binocular field; 120–130, panoramic vision; Mass and Supin (2009)	1.366 ± 0.13
*Grampus griseus*	Risso’s dolphin	2	< 24 h from stranding (CC 2)	Furutani (2008)	20–30° binocular field; 120–130, panoramic vision; Mass and Supin (2009)	1.553 ± 0.19
*Globicephala melas*	Long-finned pilot whale	1	< 24 h from stranding (CC 2)	Furutani (2008)	20–30° binocular field; 120–130, panoramic vision; Mass and Supin (2009)	1.258 ± 0.18
*Ziphius cavirostris*	Cuvier’s beaked whale	2	< 24 h from stranding (CC 2)	Furutani (2008)	20–30° binocular field; 120–130, panoramic vision; Mass and Supin (2009)	1.748 ± 0.19
*Physeter macrocephalus*	Sperm whale	1	< 24 h from stranding (CC 2)	Kojima (1951)	No binocular field; 120–130, panoramic vision; Mass and Supin (2009)	2.585 ± 0.37
Baleen whales (Mysticetes)						
*Balaenoptera physalus*	Fin whale	1	< 24 h from stranding (CC 2)	Huggenberger et al. (2019)	No binocular field; 120–130, panoramic vision; Mass and Supin (2009)	1.548 ± 0.16

The brains of marine mammals were sampled by the *Mediterranean Marine Mammal Tissue Bank* (www.marinemammals.eu) a CITES-recognized tissue bank (CITES IT 020) located at BCA that harvests and stores tissue from marine mammals that *a*) stranded along the Mediterranean and European coastline; or *b*) died in marine theme park. The brains of all terrestrial and semi-aquatic Cetartiodactyls (except those of the sheep) and those of the chimpanzees were collected during necropsy of animals that died in theme park and were delivered to BCA for postmortem diagnosis. The brains of the sheep and horses were harvested at local abattoirs during routine slaughtering of animals raised for meat production. During these latter procedures, the sheep and horses were constantly monitored by veterinary medical personnel that ensured the conditions of animal welfare requested by the current European (EC # 1099/2009; https://ec.europa.eu/food/animals/welfare/practice/slaughter_en) and Italian laws that regulate slaughtering. Finally, samples of the pig-tailed macaque are archival.

Post-mortem intervals (PMI) are noted in Table 1. From the onset of death, the process of autolysis starts to degrade biological tissues, and the brain is involved in the process. In laboratory contexts, where relatively small animals can be perfused with fixative right after euthanasia, post-mortem intervals are maintained to an absolute low. In the case of larger animals, brains harvested in the occasion of necropsies and strandings were put and kept in 4 °C refrigerators for a few hours until the necropsy. This common practice is routinely employed to avoid damaged induced by longer PMI and thus curtail substantial loss of tissue quality, and stain. The preservation status of the bodies of stranded cetaceans is classified following an internationally recognized coding system (Ijsseldijk et al. 2019, see Table 1).

### Sampling and histological techniques

The visual cortex of each species was sampled based on the topography reported in published references (see Table 1). The tissue blocks were processed for paraffin embedding and cut into thin Sections (5 µm). The resulting sections were mounted on glass slides (Superfrost plus, Menzel Gläser, Thermo Scientific, J1800AMNZ) and stained using Nissl and Klüver-Barrera techniques. Briefly, for Nissl staining, after deparaffinization in xylene and hydration in alcohol series, sections were rinsed in distilled water, dipped for 4 min in a 0.4% thionine solution, quenched in tap water, then rinsed again in distilled water, before passing in graded ethanol for dehydration, subsequent xylene baths, and coverslipped in mounting medium. For Klüver–Barrera staining, after the rehydrated sections spent a night in luxol fast blue at 57 °C, they were washed in ethanol 95% for 15 min, then in tap water for 10 min, and briefly in distilled water before differentiation in a 0.05% lithium carbonate solution for one minute. Sections passed through a 70% ethanol bath and subsequent additional lithium carbonate differentiation if the staining seemed too strong. Secondly, sections were immerged in cresyl violet 0.1% for 20 min before rinsing in tap water, graded dehydration in ethanols and ultimately in xylene, to be coverslipped.

Nissl-stained sections (three per specimen) were then scanned using a semi-automated digital microscope (D-Sight, Menarini Diagostics, Florence, Italy) at a 40 × enlargement, at the best focal plane. Within the high-resolution images acquired, we selected a large (approx. 2000 µm) straight cortical region and 3 independent operators (J-MG, AP, and BC) segregated them in layers, later analyzed separately, based on the canonical accepted structure of the striated cortex (Goebel et al. 2012). The resulting layers were then analyzed individually, for each specimen. References to large or small neurons and glia follow Garcia-Cabezas et al. (2016). The presence of granule cells, usually accompanied by slightly wider neuropil spaces, in the vicinity of lower layer 3 and upper layer 5 were critical to identify a layer 4.

### Immunohistochemistry

Immunohistochemistry was performed on paraffin Sections (8 μm). Immunoreaction was achieved using the following antibodies against calcium binding proteins (CBPs): a mouse monoclonal anti-parvalbumin (PV, 1:2000, Swant), a rabbit polyclonal anti-Calbindin D-28 k (CB, 1:1000, Swant), a mouse anti-Calretinin (CR, 1:1000, Swant) (see Table 2). Antigen retrieval was carried out at 90 °C for 10 min in a 0.05 M Tris/HCl solution buffer, pH 9.0. To block endogenous peroxidase activity, sections were treated with a 1% H2O2 solution in Tris-buffered saline (TBS, 150 mM NaCl, 50 mM Tris–HCl, pH 7.6) for 10 min. Sections were then incubated for 1 h at room temperature, then overnight at 4 °C in a solution containing the primary anti-PV, anti-CB or anti-CR in SuMi (0.5% Triton X-100 and 0.25% gelatin in TBS). Sections treated with rabbit antibodies were then rinsed in TBS (3 × 5 min), and incubated in biotinylated anti-rabbit IgG (5 μg/ml, Vector Labs, Burlingame, CA) diluted 1:400 in SuMi for one hour at room temperature. After rinsing in TBS, sections were incubated in 1:800 avidin–biotin complex (Vectastain Kit, PK-7200, Vector Labs, Burlingame, CA) for an hour. Sections were again rinsed in TBS. Staining was revealed by incubating the sections in a diaminobenzidine solution with 0.2% nickel ammonium sulfate and 0.01% hydrogen peroxidase. Sections with mouse primary antibodies were treated using a horseradish peroxidase reaction, first with a FLEX/HRP labeled polymer, revealed by a diaminobenzidine–chromogen solution (EnVision FLEX system, Dako). Subsequently, all sections were counter-stained with thionine for 4 min, then dehydrated in graded ethanol solutions (3 min each) and finally in xylene before glass coverslipping with mounting medium.

**Table 2:** Primary antibodies used in the present work

Antibody	Immunogen	Manufacturer’s details	Dilution
Anti-PV	Produced by hybridization of mouse myeloma cells with spleen cells from mice immunized with parvalbumin purified from carp muscles	Swant, mouse monoclonal, Code No: 235, Lot no: 10–11 (F) RRID: AB_10000343 (https://antibodyregistry.org/AB_10000343)	1:1000
Anti-CR	Produced in mice by immunization with recombinant human calretinin − 22 k (identical with calretinin up to Arg178 N-terminal)	Swant, mouse monoclonal, Cat# 6B3, Lot n° 010,399 RRID: AB_10000320 (https://antibodyregistry.org/AB_10000320),	1:1000
Anti-CB	Produced against recombinant rat calbindin D-28 K (CB)	Swant, rabbit polyclonal, Lot No.: 9.03, Code No.: CB − 38a RRID: AB_10000340 (https://antibodyregistry.org/AB_10000340)	1:1000

Negative controls were performed by replacing one of the steps with TBS or non-immune serum, in which case staining was abolished. Positive controls were carried out testing the primaries antibodies on mouse brain sections. Additionally, specificity of the antibodies had already been tested in previous studies (see Table 2).

### Image analyses

Shortly, the image analysis process was performed on the Nissl-stained material using space-varying thresholds to identify all the cell bodies stained by thionine in the area and separate them from the background. Cell clusters were separated by identifying cell centers by local density estimation, layer ordering constraints based on the object values of eccentricity, areas and solidity (Grisan et al. 2018). The resulting populations of cells were then analyzed through Matlab function algorithms (Matlab, Mathworks, Natick, MA, USA), identifying cell area, perimeter, and shape descriptors circularity, solidity, extent and a surface density (number of cells in a radius of 50 µm for any given cell). Given the thickness of the Sections (5 µm), no overlapping of cells could be expected, however an estimation of volume density would overestimate the number of neurons (for details see Corain et al. 2020). This measure allows us to perform comparisons across brains of different sizes, and implicitly assess how neuron count and neuron size is scaling with the brain volume.

Cortical thickness was measured using the calibrated software provided for the automated microscope (Navi Viewer, Visia Imaging, San Giovanni Valdarno, Italy), using a tool measuring the length perpendicular to a line placed on the pial surface of the cortex. Measurements were made at least 5 times per sample, outside of sulcus bottom or top to avoid distortions.

### Statistical analysis

The same approach has been used for every grouping variable, that is the species, the phylogenetic grouping and the orbit orientation, as it was used in previous works (Corain et al. 2020).

The comparison of the morphometric descriptors (**Y**) among the groups has been formalized by the following statistical linear model

1 $${\mathbf{Y}}_{ij} = {{\varvec{\upmu}}} + {{\varvec{\uptau}}}_{j} + {{\varvec{\upvarepsilon}}}_{ij} ,$$

where specific location ***τ***_*j*_ for the group *j* can be a random variable (i.e. not a fixed effect) and scale effects ***σ***^2^(*τ*_*j*_) = ***σ***^2^_*j*_, are both allowed to differ across populations under the alternative hypothesis, while the random components **ε**_*ij*_ are assumed to be the same for each observation—at least under the null hypotheses–but are not specified in their distributional form according to a nonparametric permutation-oriented approach (Pesarin 2001; Arboretti et al. 2014).

Under the null hypothesis the observations of the groups are assumed to be equal, that is the random variable ***τ***_*j*_ is assumed to follow the same distribution in each group.

Permutation-based *p* values (Pesarin 2001; Corain and Salmaso 2015) have been calculated under the null hypothesis of exchangeability.

The tests of the same domains (size, shape and density) are further combined in a multivariate test by the Fisher combining function in order to assess the equality in distribution of the groups in a multivariate perspective.

Finally, pairwise test have been performed through constrained permutation among the two groups and using the same method described above. Furthermore, Shaffer post-hoc correction for multiple comparison was performed.

The R package “flip” together with custom codes were used to run the analysis (Finos 2018).

### Limits of the present study

The present study describes domestic but also rare and wild species. The low number of specimens for some of the latter species is a limit. Statistically, the potentially comparative high value of the quantitative results provided here is hindered by the small number of specimens (*n* = 1 for some). Furthermore, tissues were collected through the years, and sampling conditions in the field may have affected Klüver–Barrera stain of myelin sheaths, and in rare cases a diminished immunoreactivity. Nonetheless, even considering these limitations, the statistical weight of the semi-independent analysis of a large cohort of cells in each sample (see below) should alleviate some of this impact. Extrapolation of some of our absolute numbers to another context may challenging. However, their analysis maintains a comparative significance if referred to species often largely overlooked or never analyzed before in detail. We also emphasize that density is absolute, not relative to body size or cortical thickness, and uncorrected for processing.

## Results

### Overall quality of the tissues

**Fig. 1:**
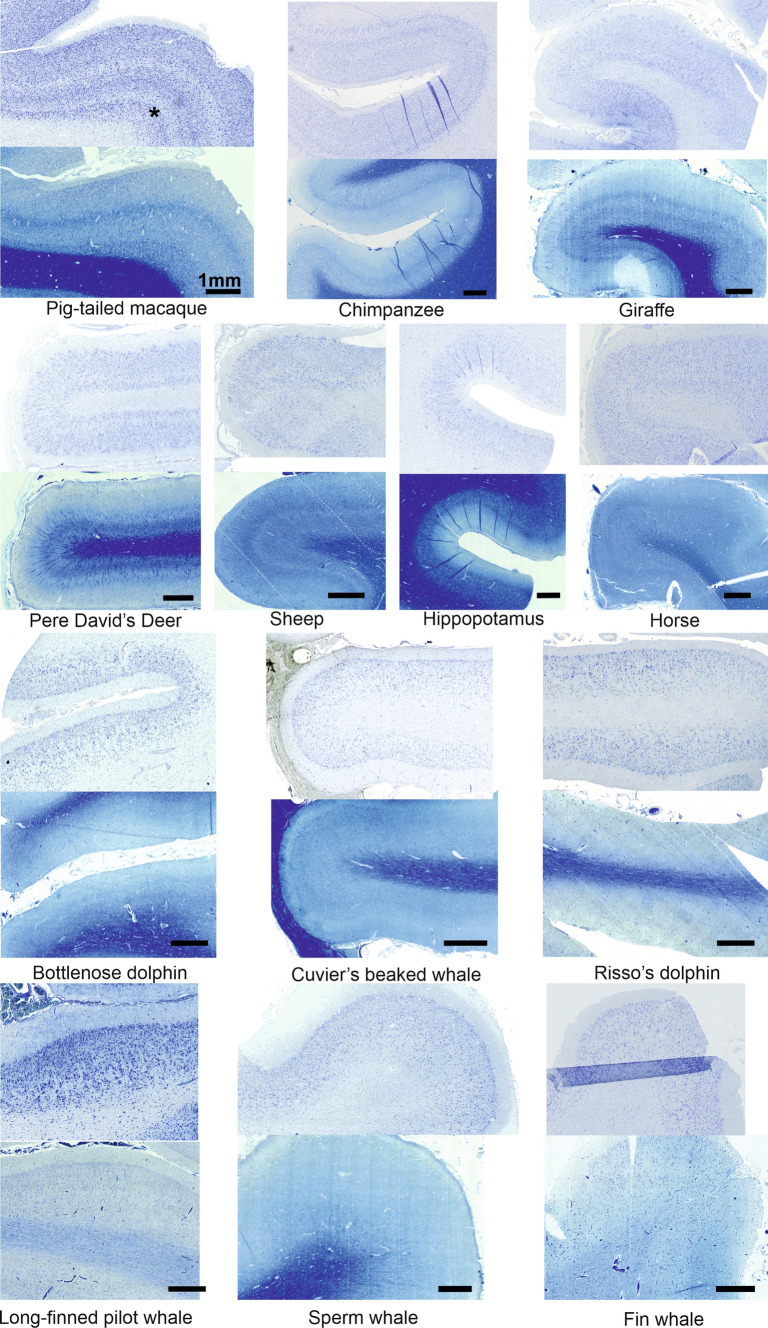
Microphotographs of Nissl and Klüver–Barrera tissue staining of primary visual cortices of selected species. The clear pattern of the Gennari line seen in the macaque is also very clear in the giraffe, which heavily relies on vision. It is also noticeable in the deer and sheep, but is not equally present in aquatic mammals. In the bottlenose dolphin, a hint of myelin double band is visible. This is less true in Cuvier’s beaked whale, and unnoticeable in the fin whale. The quality of the tissue might negatively influence myelin staining of the Klüver–Barrera in some cetacean species. All bars are 1 mm

All the examined samples yielded results of comparable visual and technical quality. Layering, although in different degrees, was evident in all species (see below) (Fig. 1).

### Cortical thickness

See (Fig. 2).

**Fig. 2:**
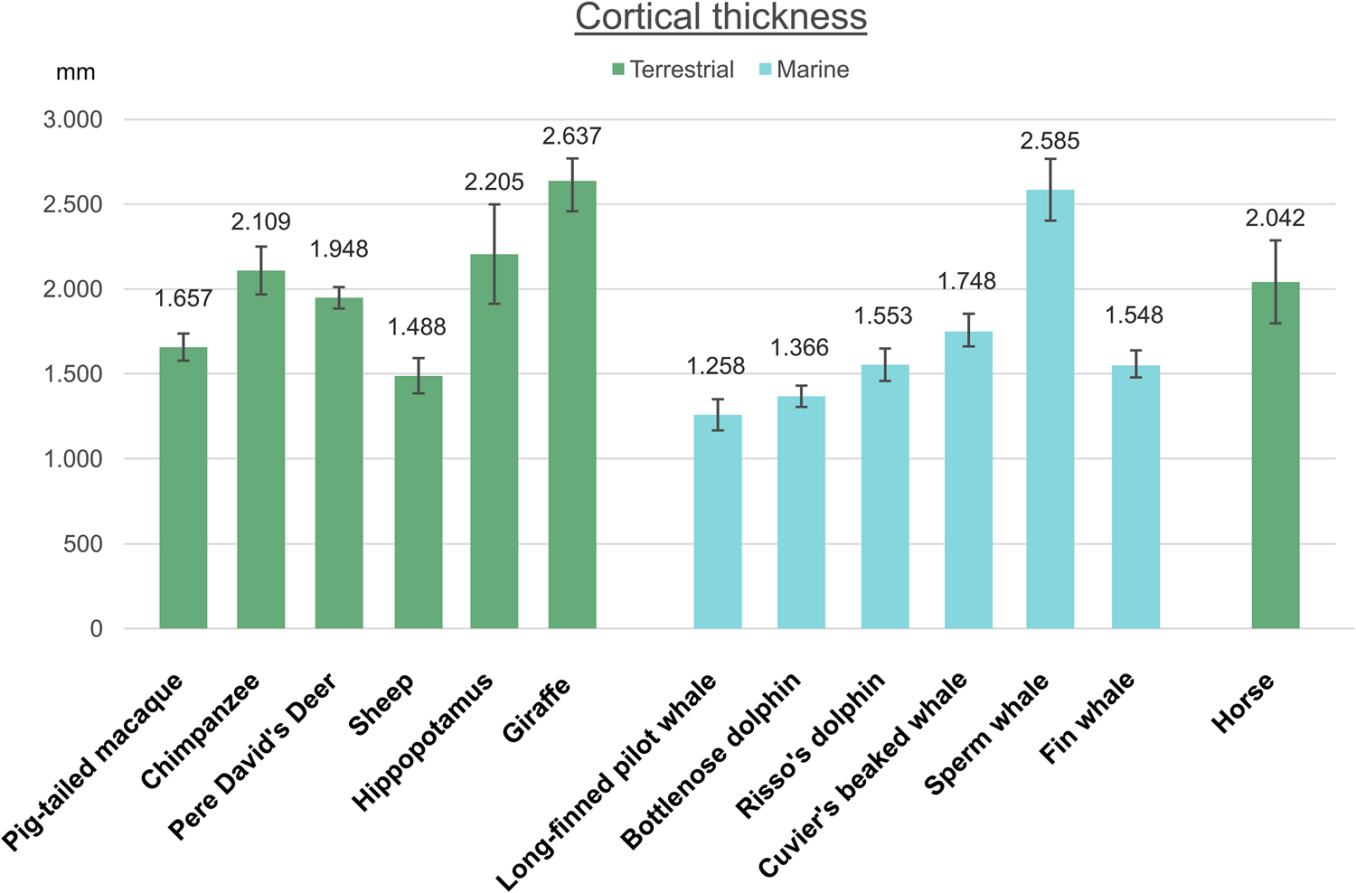
Graphical representation of the cortical thickness measurement means. The values are in mm, the error bars represent the standard deviation

#### Lamination and immunocytochemical results per species

Overall, most species maintained a distinction in recognizable layers in the lower part of the cortical columns, consistent with the accepted model for V1 (Niebur and Wörgötter 1994). A notable exception was the hippopotamus where the columns were impossible to recognize.

### Primates

#### Pig-tailed macaque (*Macaca nemestrina*)

**Fig. 3:**
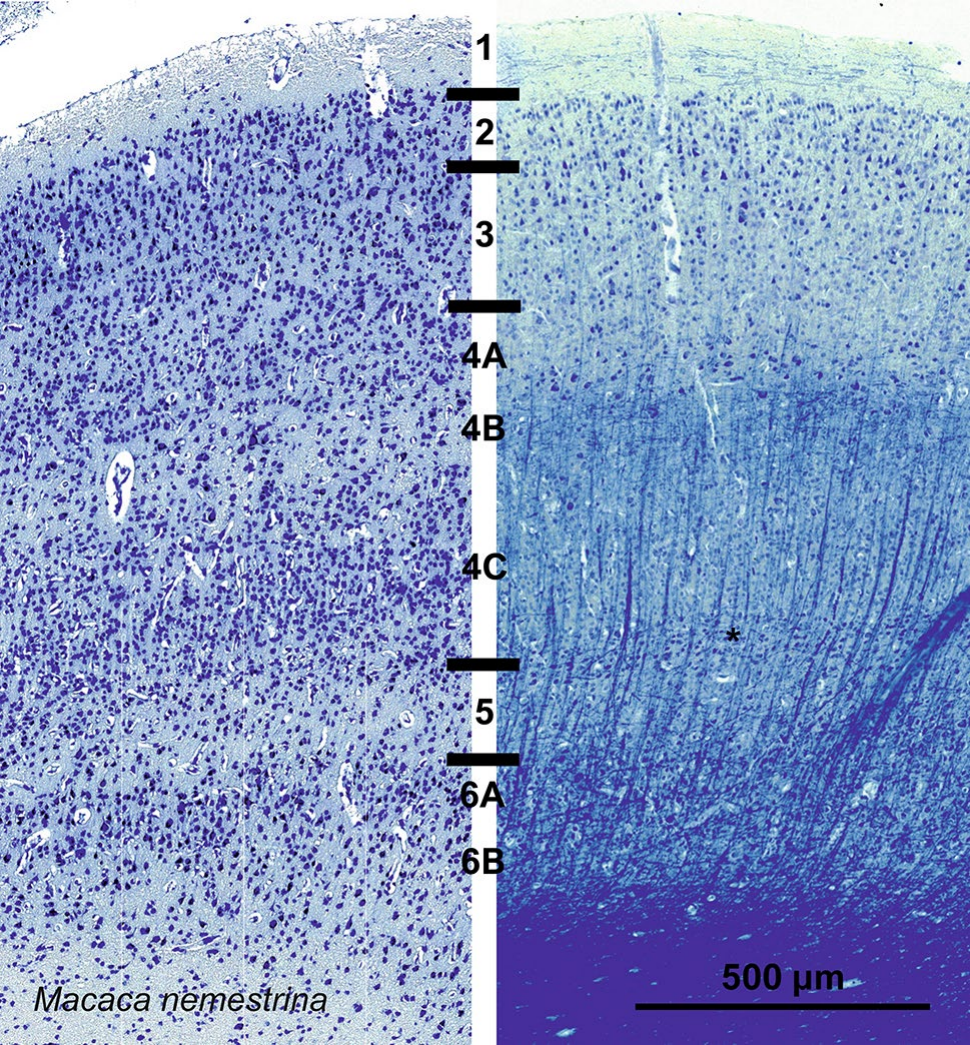
Photomicrograph of Nissl (left) and Kluver–Barrera (right) stain of the primary visual cortex of the pig-tailed macaque (*Macaca nemestrina*). The images have been voluntarily slightly overexposed to enhance the structure visualization

The visual cortex of the Rhesus macaque (*Macaca mulatta*) has been the reference in the field and was used as basis for comparison in this study, although our tissue samples are those of a closely related species, *Macaca nemestrina*. The details of its structure have been extensively described elsewhere (Peters 1994; Morrison et al. 1998), and we will only briefly summarize them here. The first layer was composed of most of the apical dendrites of inner layers and small stellate neurons. L2, together with L3, although denser, contained medium and small pyramidal cells as well as stellate cells. The lower border with L4A was relatively difficult to find, marked by dense round cells. The cell poor L4B was easily identified, containing large Meynert cells. L4C in macaque could be subdivided into a L4Ca and a L4Cb, the latter being somewhat denser and more obviously organized in vertical columns. The fifth layer was cell-sparse, mainly composed of pyramidal cells of medium size, with few solitary Meynert cells. L6 was also subdivided in two sub-laminae, the external 6A containing packed pyramidal cells and solitary cells of Meynert, and the inner 6B facing white matter, with sparse neurons (Fig. 3).

**Fig. 4:**
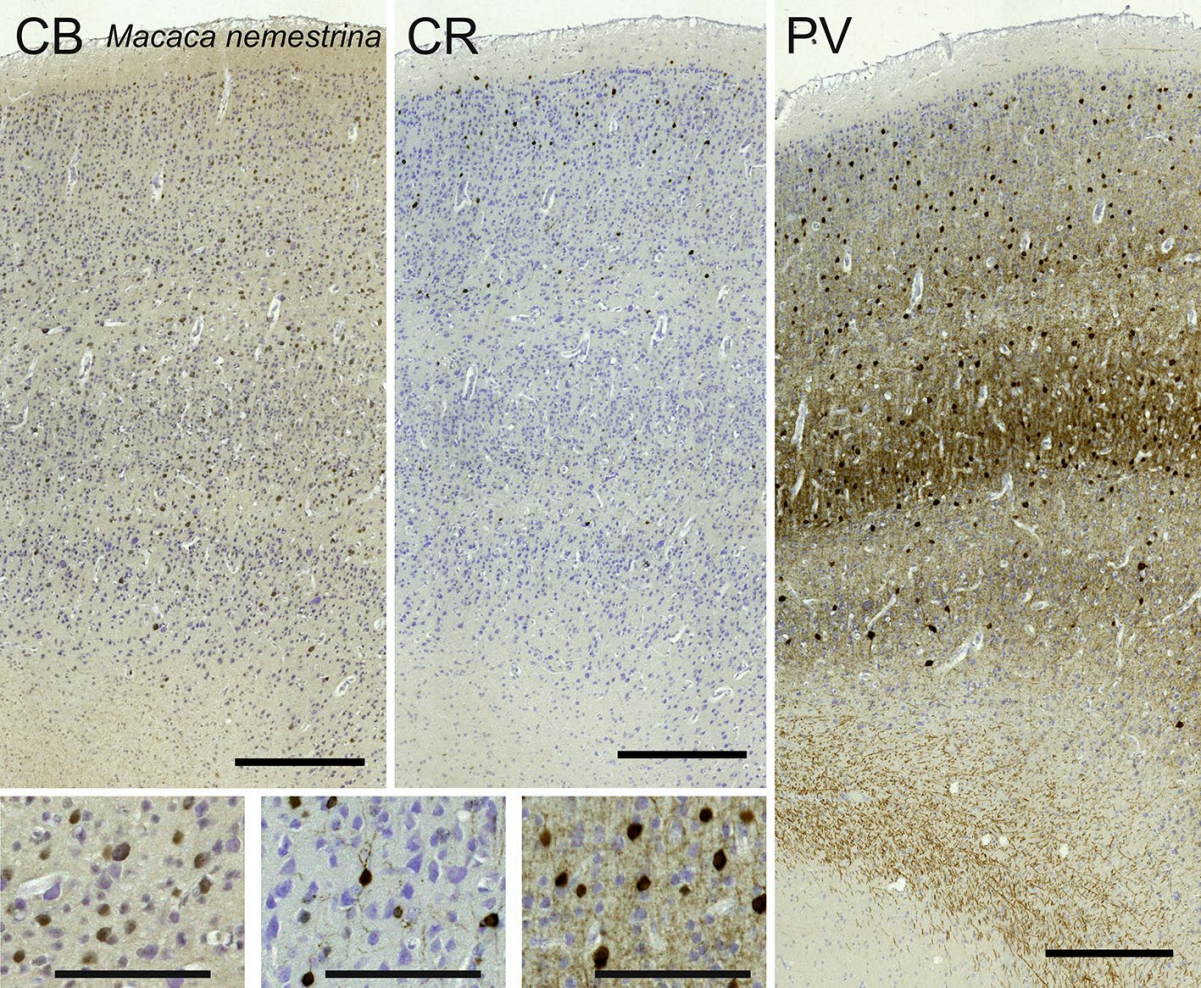
Microphotographs of CB-ir (left), CR-ir (middle) and PV-ir (right) neurons in the primary visual cortex of the pig-tailed macaque (*Macaca nemestrina*), with magnified inserts below. Bars are 300 μm, 100 μm in inserts

Calbindin-immunoreactive (-ir) neurons were found throughout the cortical thickness of the macaque visual cortex, within pyramidal and non-pyramidal neurons. A higher number could be seen in the external layers, although not as prominently as expected (Fig. 4). In layer 2 and 3, the majority of cells were relatively small, multipolar, with no or very short staining of the axons and dendrites. In lower layers (L4 to L6), larger round cells were also found, with at least two poles, one ascending to higher layers, the others more laterally. Finally, small round multipolar cells were seen in layers L2 to L6, with thin dendrites. Calretinin neurons were found primarily in layers L1, L2 and L3 with vertically oriented fusiform bodies and long dendrites emerging from the vertical poles (Fig. 4). Some triangularly-shaped neurons were found in L5 and L6, their ascending dendrite reaching higher layers. In the macaque, the arborization and fibers in general were less evident than in the rest of our preparations. Parvalbumin immunoreaction was most represented in layers L3 to L5. A first kind of large multipolar cells were seen, with projections mainly directed horizontally. Vertical fibers were obviously present, their density marking two dark bands in layers L4A and L4C (Fig. 4).

#### Chimpanzee (*Pan troglodytes*)

**Fig. 5:**
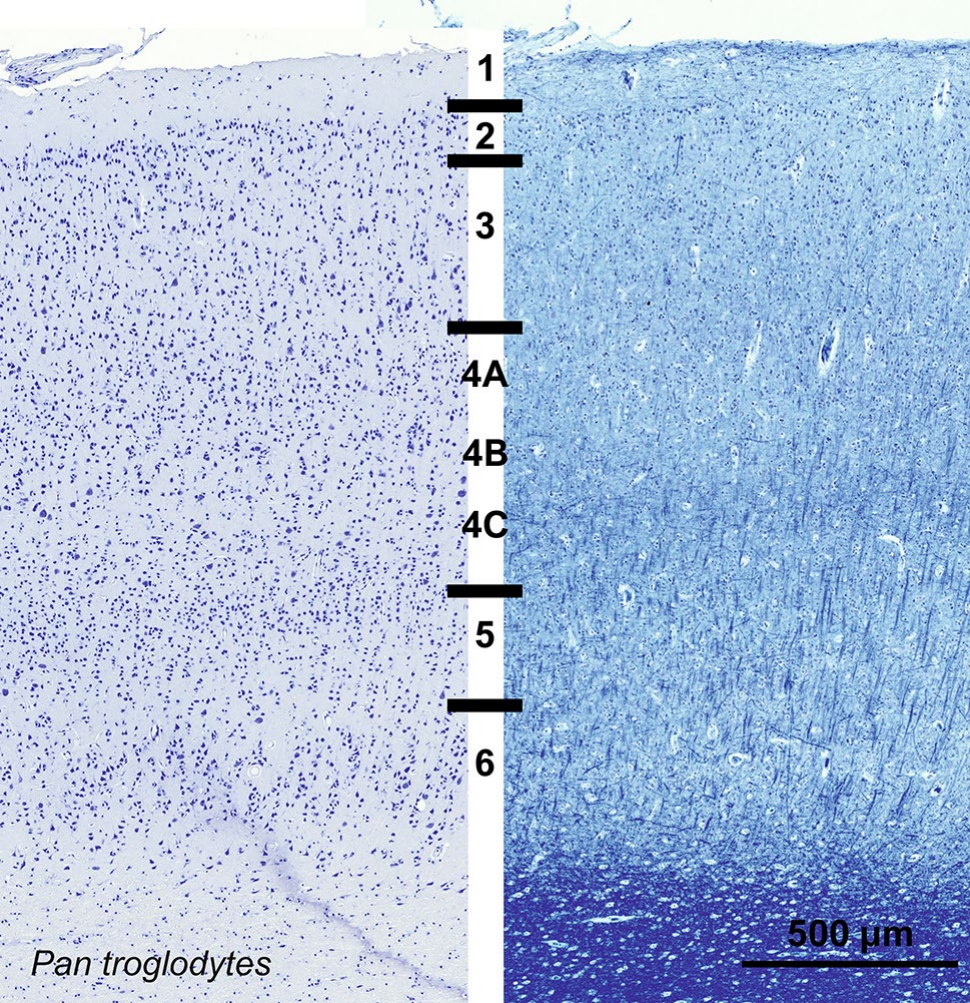
Photomicrograph of Nissl (left) and Kluver–Barrera (right) stain of the primary visual cortex of the chimpanzee (*Pan troglodytes*). The images have been voluntarily slightly overexposed to enhance the structure visualization

**Fig. 6:**
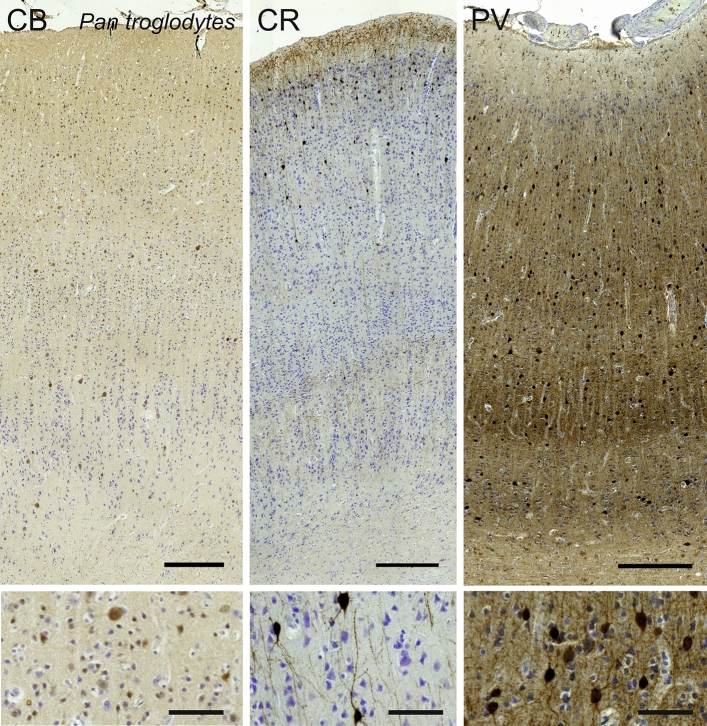
Microphotographs of CB-ir (left), CR-ir (middle) and PV-ir (right) neurons in the primary visual cortex of the chimpanzee (*Pan troglodytes*), with magnified inserts below. Bars are 300 μm, 100 μm in inserts

The visual cortex of *Pan troglodytes*, much thicker than that of the macaque, displayed a similar pattern with easily identified canonical primate features and a more obvious “rain drop” pattern than in the macaque (Fig. 5). The large multipolar CB-ir cells seemed sparser, located in L4A, L5 and L6, while a population of small multipolar and bipolar cells were found in L2–L3, and in fewer numbers in L4A and B (Fig. 6). CR-ir cells were located similarly in L2–L3, but had a much clearer bipolar type with their dendrites arranged in a vertical column (Fig. 6). A large number of fibers were also present in L1, together with few somata. A lesser band of fibers was clearly demarcated at the junction between L4C and L5, with occasional small round cells. In the chimpanzee, parvalbumin was found in neurons in L3 to L6, the majority of which were large multipolar neurons with dendrites extending mostly laterally with an ascending fiber. Dark fibrous bands in L4A and L4C could also be seen (Fig. 6).

### Perissodactyls

#### Horse (*Equus caballus*)

**Fig. 7:**
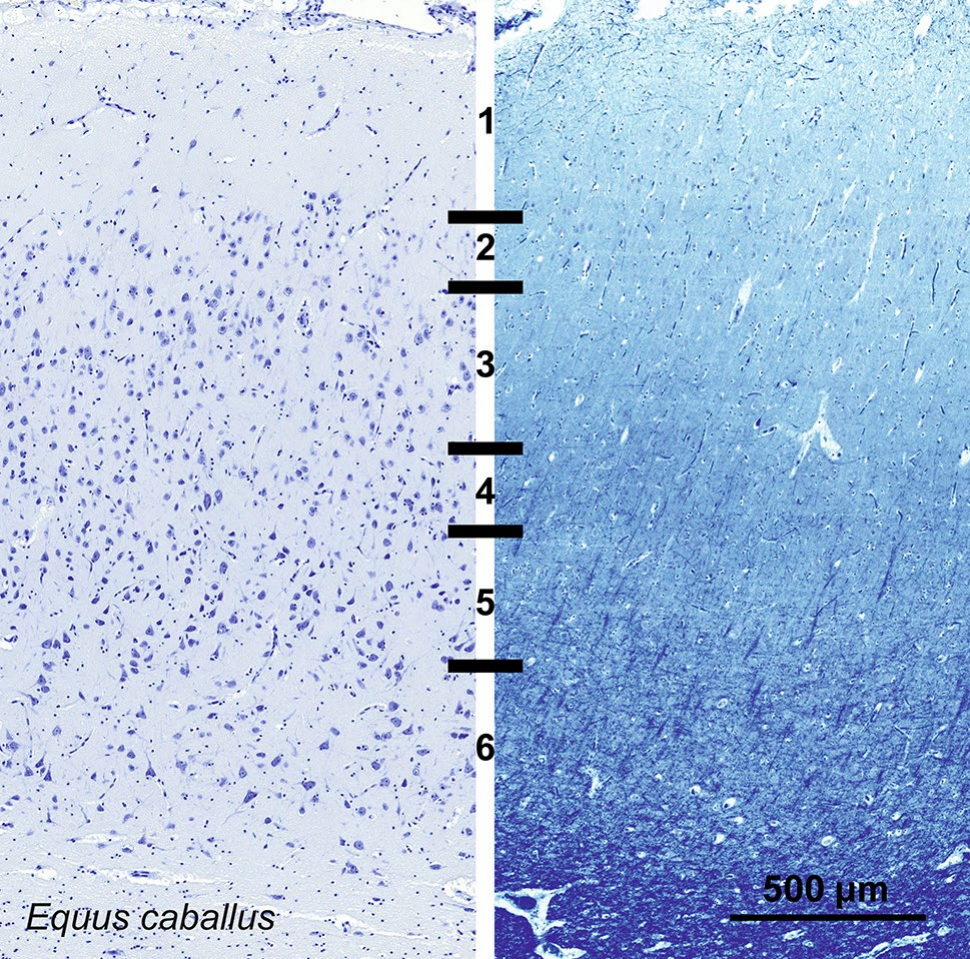
Photomicrograph of Nissl (left) and Kluver–Barrera (right) stain of the primary visual cortex of the horse (*Equus caballus*). The images have been voluntarily slightly overexposed to enhance the structure visualization

The visual cortex of *Equus caballus* showed a relatively thick L1, undifferentiated L2 and L3 with relatively large neurons in the upper L3. A group of smaller dense neurons together with a myelin slightly darker band suggested a putative L4. Raindrops columns in L5 with darker pyramidal neurons and larger cells in L6 were traversed by some short vertical bundles reaching the top border of L5 (Fig. 7).

**Fig. 8:**
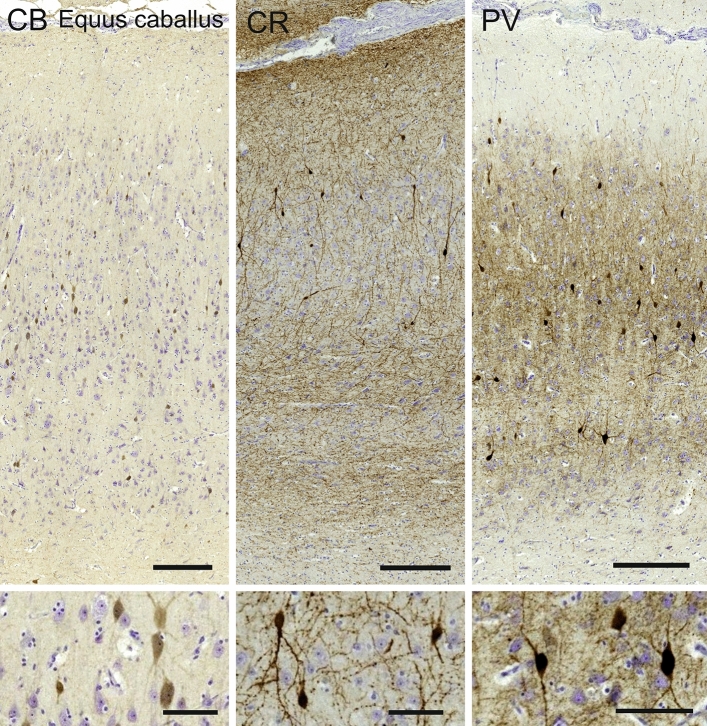
Microphotographs of CB-ir (left), CR-ir (middle) and PV-ir (right) neurons in the primary visual cortex of the horse (*Equus caballus*), with magnified inserts below. Bars are 300 μm, 100 μm in inserts

Calbindin-ir cells were bipolar and vertically oriented, in majority found in the upper L5 but present in L3. Dendrites reached L2 and L1. Few cells were also marked in L6 (Fig. 8). Calretinin-positive neurons were mostly present in upper L2 to lower L3, showing long dendrites reaching L1. Fewer, smaller perikarya were seen in L5. Varicose nets were seen mostly in pial L1 but two bands were present in most of L5 and lower L6 at the white matter border (Fig. 8). Parvalbumin showed a much more diffuse reactivity in the horse V1. The multipolar larger cells were present in lower layers L6 and L5 while most of the bipolar cells were slightly smaller and present in L3 to L2. Dendritic fibers reached most of the cortical thickness except in L1, lower L6 and a thin band in lower L5 (Fig. 8).

### Terrestrial Cetartiodactyls

#### Sheep (*Ovis aries*)

**Fig. 9:**
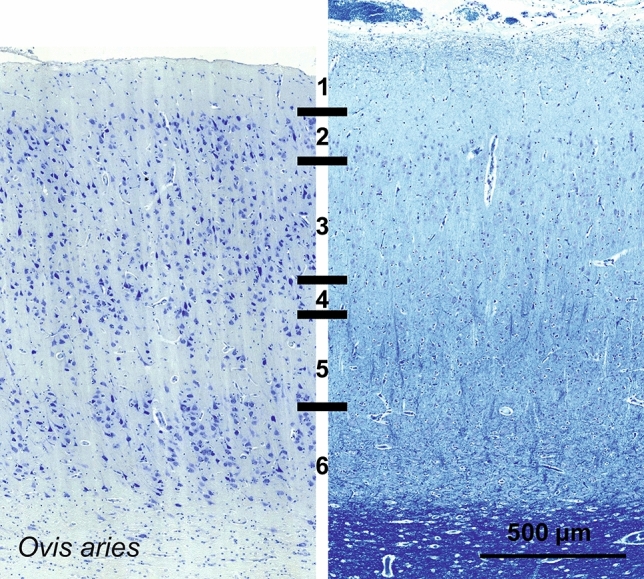
Photomicrograph of Nissl (left) and Kluver–Barrera (right) stain of the primary visual cortex of the sheep (*Ovis aries*). The images have been voluntarily slightly overexposed to enhance the structure visualization

The V1 area of *Ovis aries* was marked by a relative lesser distinction of pyramidal vs. granular cells comparatively to that of primates. The molecular layer (L1) was relatively medium-sized. Below were a relatively thin L2 and L3, a notable L4 containing some granular cells, a thin L5 marked by large pyramidal neurons and a wider L6. Throughout the cortical thickness, relatively large somata, mostly triangular in shape (Fig. 9) with their major axis oriented radially, were organized in columns, interspersed with myelin bundles reaching out to a faint if present L4. The sublamination of L4 was not clearly apparent, but sparse granular cells could be spotted. Layers L1 and L6 appeared relatively thick, the L2-L3 boundary was not easily identified.

**Fig. 10:**
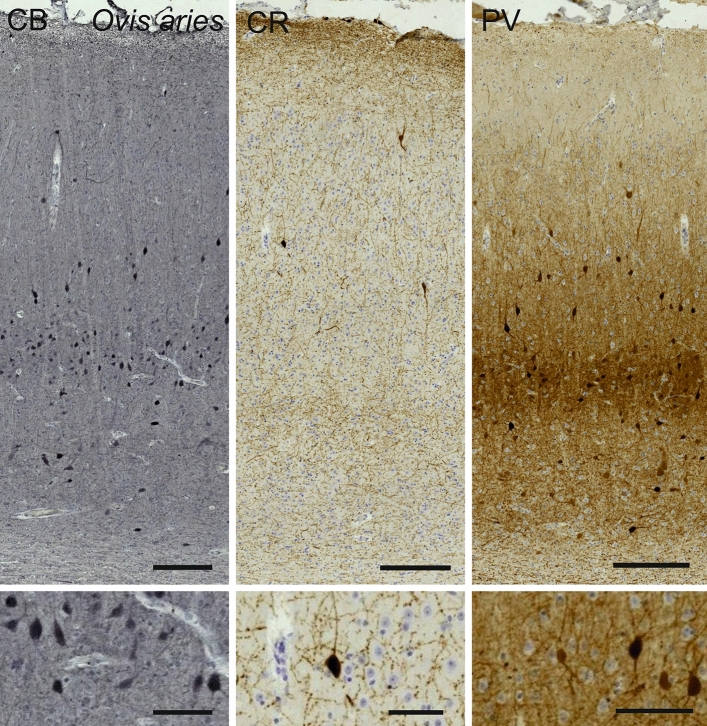
Microphotographs of CB-ir (left), CR-ir (middle) and PV-ir (right) neurons in the primary visual cortex of the sheep (*Ovis aries*), with magnified inserts below. Bars are 300 μm, 100 μm in inserts

Calbindin labeling highlighted mostly bipolar cells in L2-3 and 5, some multipolar stellate cells in L5 and 6 and occasional large cells projecting radially in L6 (Fig. 10). Calretinin was found in few cells in the sheep, the wide majority of which were bipolar radially oriented interneurons located in L5 and 6. A faint fiber band was present along L6 and clearer along the L1 pial surface (Fig. 10). Parvalbumin-ir cells appeared relatively large and round with long dendrites extending clearly to L3 and L2 pyramidal neurons (Fig. 10). Some longitudinally oriented cells were found in L6, but the majority of the perikarya were centered on L5, where a dark dendritic fiber band was seen.

### Père David’s deer (*Elaphurus davidianus*)

**Fig. 11:**
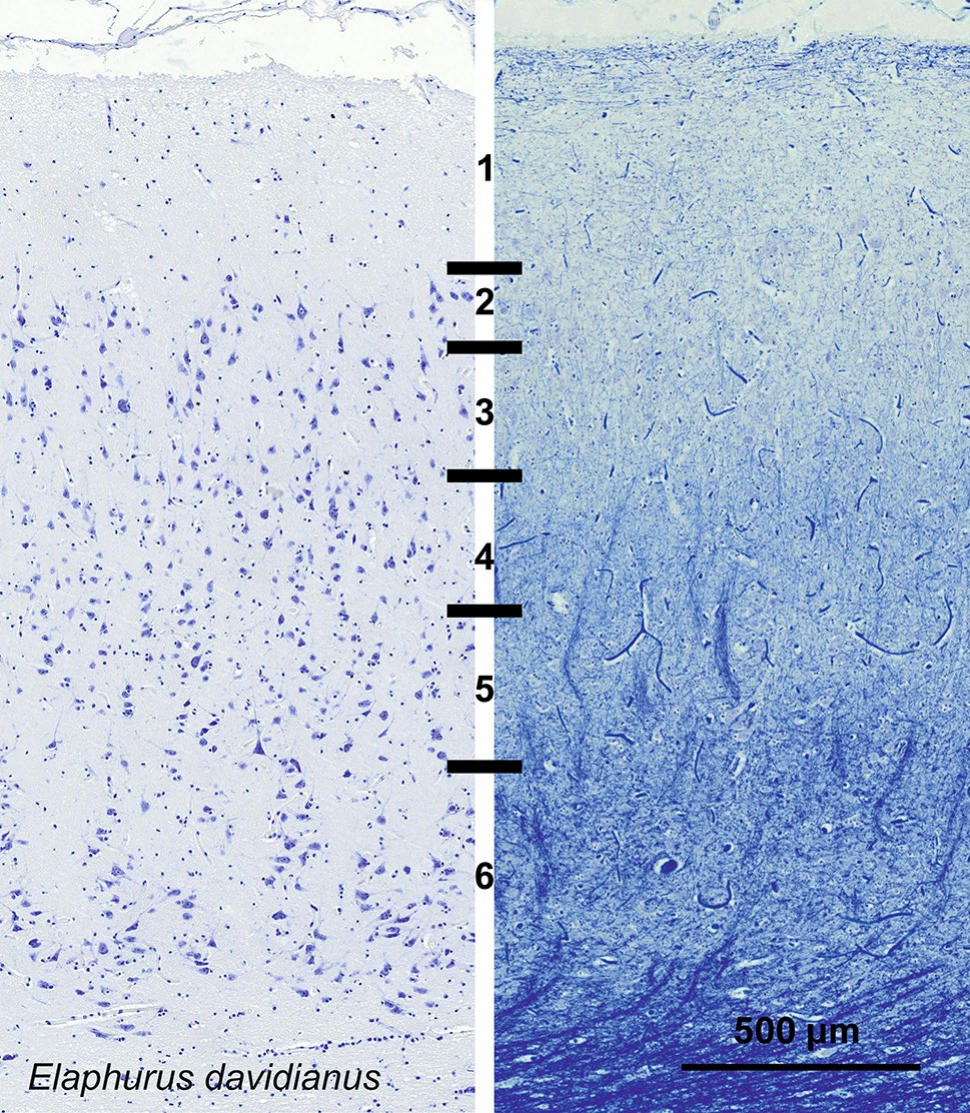
Photomicrograph of Nissl (left) and Kluver–Barrera (right) stain of the primary visual cortex of the Père David’s deer (*Elaphurus davidianus*). The images have been voluntarily slightly overexposed to enhance the structure visualization

**Fig. 12:**
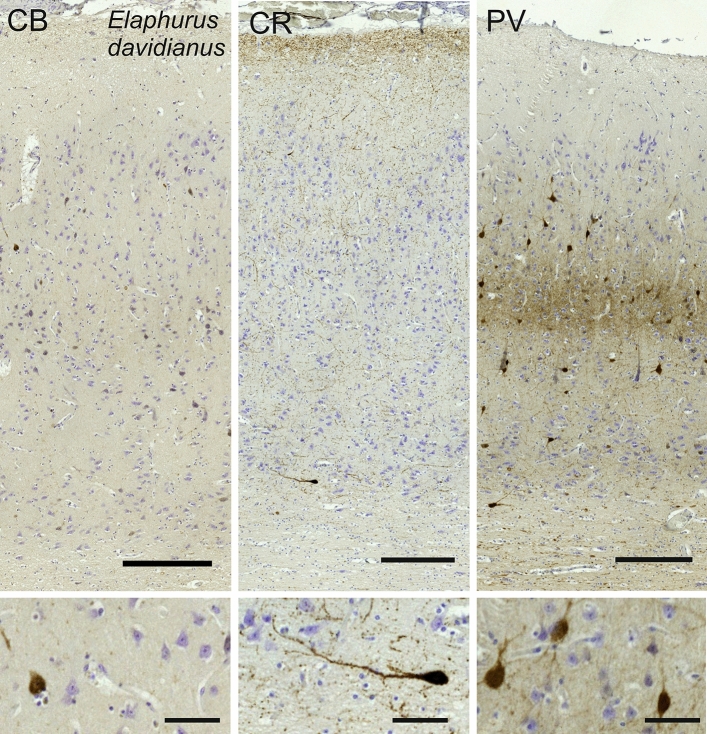
Microphotographs of CB-ir (left), CR-ir (middle) and PV-ir (right) neurons in the primary visual cortex of the Père David’s deer (*Elaphurus davidianus*), with magnified inserts below. Bars are 300 μm, 100 μm in inserts

The V1 of *Elaphurus davidianus* had a primary visual cortex very similar to that of the sheep (Fig. 11, 12) although PV-ir fibers were seemingly forming a higher neuropil band and multipolar cells were present higher in L3 (Fig. 12).

### Giraffe (*Giraffa camelopardalis*)

**Fig. 13:**
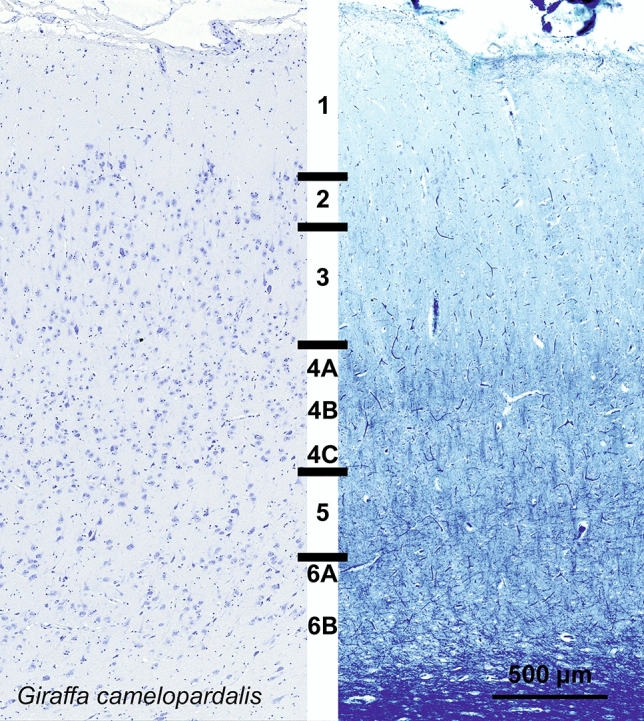
Photomicrograph of Nissl (left) and Kluver–Barrera (right) stain of the primary visual cortex of the giraffe (*Giraffa camelopardalis*). The images have been voluntarily slightly overexposed to enhance the structure visualization

The V1 of *Giraffa camelopardalis* showed a relatively thick L1, together with seemingly developed inner layers, notably L4. L2 appeared scarce compared with other species. L3 showed an apparent reduction, while L4A included numerous small granule cells (Fig. 13). The presence of myelin on the Klüver–Barrera stain showed the presence of a L4B, populated by middle to small sized neurons. L4C, was particularly difficult to pinpoint, and comported medium-sized pyramidal neurons in a denser pattern than L5 while myelin was less present. L5 presented medium and large-sized neurons with a string-like organization interspersed with neuropil columns. L6A can clearly be seen, marking the cell-dense lower strip of Gennari, with rather small pyramidal cells, and below, L6B showing a much sparser cell density, surrounded by heavy myelin tracts (Fig. 13).

**Fig. 14:**
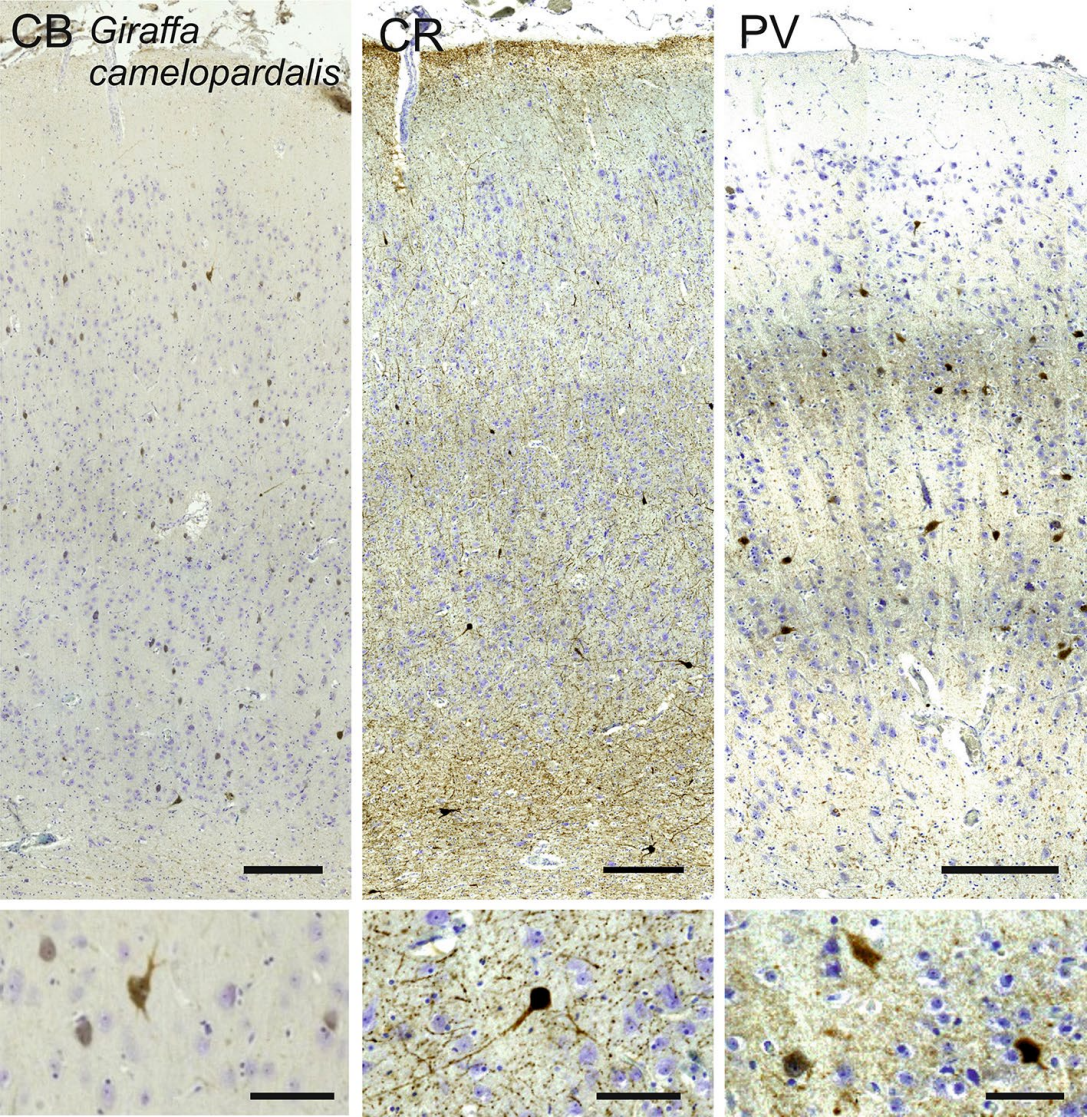
Microphotographs of CB-ir (left), CR-ir (middle) and PV-ir (right) neurons in the primary visual cortex of the giraffe (*Giraffa camelopardalis*), with magnified inserts below. Bars are 300 μm, 100 μm in inserts

CB-positive interneurons could be seen throughout L2, L3 and L5 and was notably scarcer in L4 (Figure giraffe CB). Most notable was a darker band of CB-positive fibers in L4A. The occipital sample showed a very similar result, with a wide L4 and ample dark band on the CB immunocytochemical stain (Figure giraffe occ). PV-ir neurons were found in lower L3 to upper L6 mostly, in the form of multipolar round neurons predominantly with radially oriented dendritic cones (Fig. 14) a band of PV-ir neuropil was clearly seen covering most of L4. CR-ir neurons were relatively few, mostly fusiform bitufted cells, distributed among layers, with some notable multipolar ones, not limited to L5 or L3. Their dendrites formed a band in layer L4B and L4C (Fig. 14).

### Semi-aquatic Cetartiodactyls

#### Hippopotamus (*Hippopotamus amphibius*)

**Fig. 15:**
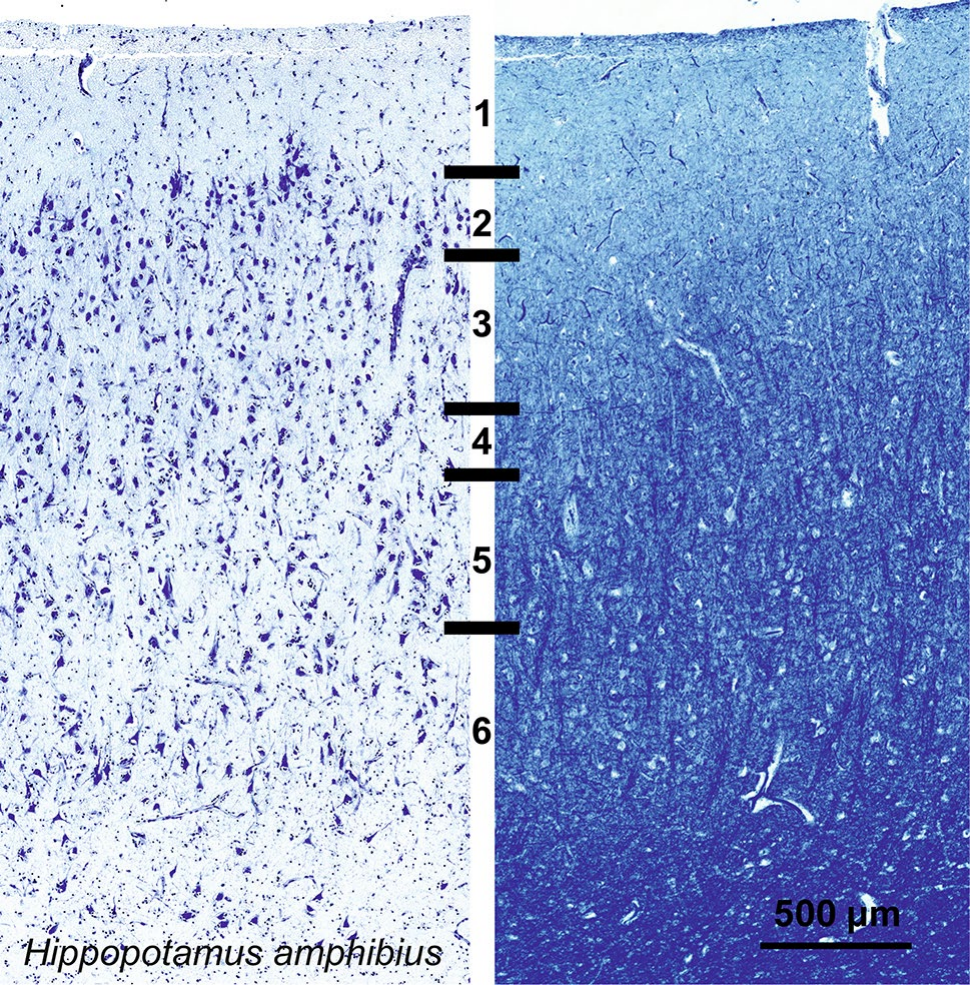
Photomicrograph of Nissl (left) and Kluver-Barrera (right) stain of the primary visual cortex of the hippopotamus (*Hippopotamus amphibius*). The images have been voluntarily slightly overexposed to enhance the structure visualization

The V1 of *Hippopotamus amphibious* showed signs of more reduced lamination in both Nissl stain and Klüver–Barrera, diminishing the structure expected in visual cortices (Fig. 15). The overall lamination was not well differentiated as the cellular components appeared more homologous across layers. In particular, L1 was rather thick, L2 cells that are typically very dense and rather small were found to be much larger and particularly hard to separate from L3 (Fig. 15). Beyond the reduction of L4, large L5 and L6 were present, with thick myelin bundles reaching as high as lower L3.

**Fig. 16:**
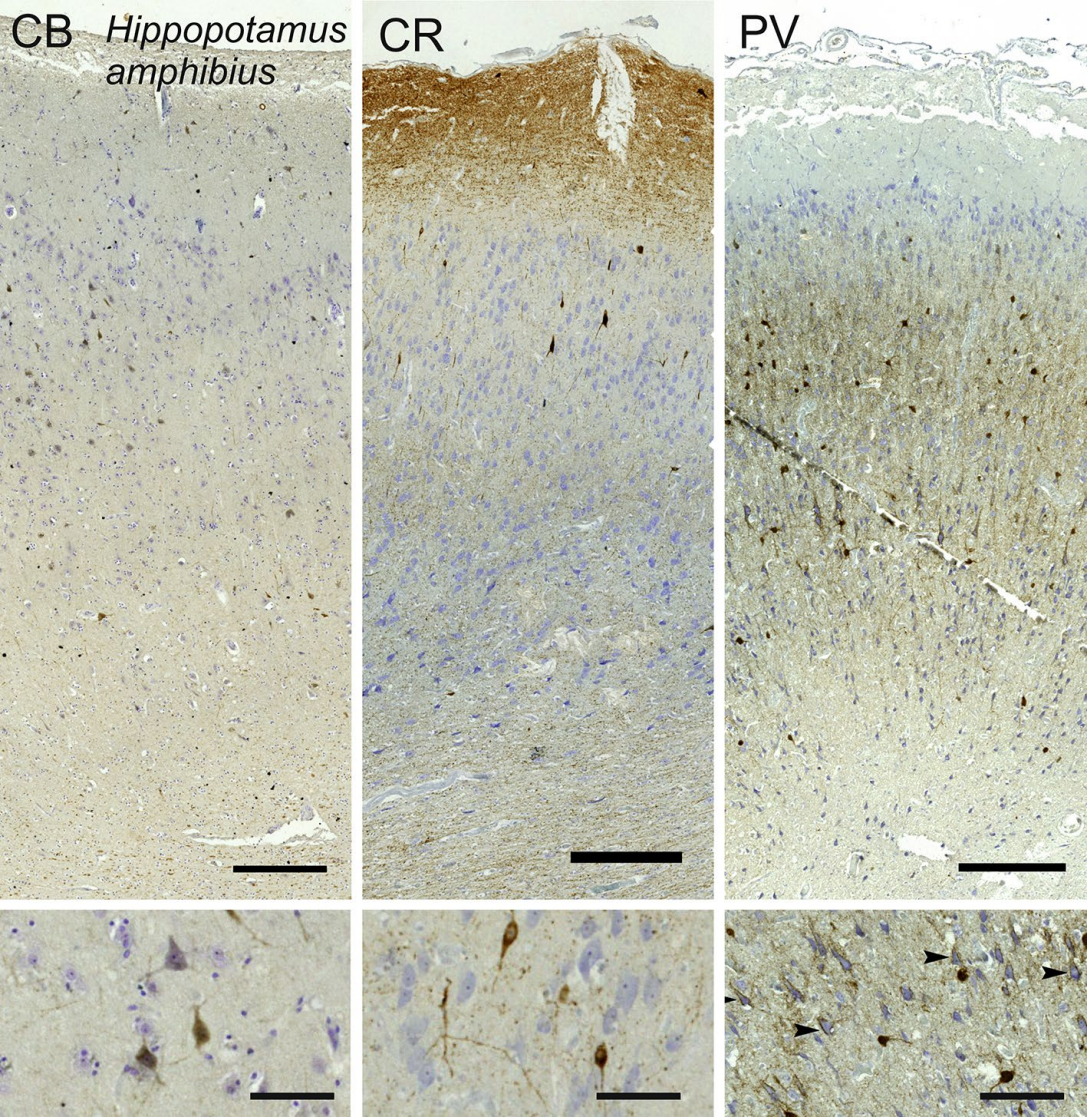
Microphotographs of CB-ir (left), CR-ir (middle) and PV-ir (right) neurons in the primary visual cortex of the hippopotamus (*Hippopotamus amphibius*), with magnified inserts below. Bars are 300 μm, 100 μm in inserts

Calbindin signal was found in middle to large sized neurons seemingly in all layers and were relatively scarce (Fig. 16). Large multipolar somata were more angular than that of other terrestrial mammals, while smaller neurons presented a more classical bipolar to multipolar pattern. Calretinin-containing neurons were scarce, and mostly located in L2-L3, with a fusiform shape and vertically oriented dendrites. A very weak fiber band could be seen in L5, while L1 had a much clearer fiber web, with small cells located near the pia (Fig. 16). Multipolar PV-ir neurons were mostly centered in L3 and L5, with fewer cells in L6, with dendrites extending mostly laterally. Besides well-stained somata, some large neurons were present with what appeared to be fiber beads arriving on their cell wall (Fig. 16 insert).

### Marine Cetartiodactyls

The overall appearance of the visual cortex was very similar in all cetaceans. V1 was very thin (approx. 1.2–1.7 mm) and showed no obvious vertical columnar organization, much like other areas. The main characteristic was the homogenous presence of large to middle-sized irregular pyramidal cells arranged in varying levels of density along the cortical layers.

### Bottlenose dolphin (*Tursiops truncatus*)

**Fig. 17:**
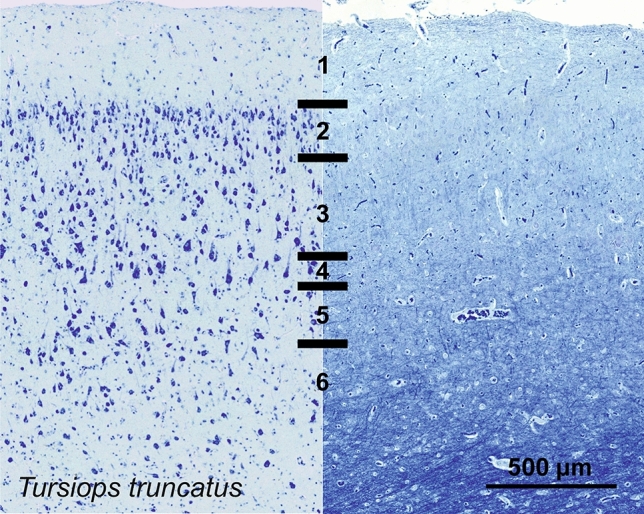
Photomicrograph of Nissl (left) and Kluver-Barrera (right) stain of the primary visual cortex of the bottlenose dolphin (*Tursiops truncatus*). The images have been voluntarily slightly overexposed to enhance the structure visualization

The V1 of *Tursiops truncatus* was typical of the cetacean cortex. L1, L2 and L3 were most prominent, L1 showing some myelinated fibers on the very pial surface, and few round cells. L2 and L3 were composed of pyramidal cells, with L2 showing the highest density of cells in the whole column, comprising mostly smaller pyramidal cells and bipolar cells (Fig. 17). L3 displayed a much more spread out pattern, with a distinct gradient from smaller to larger pyramidal cells along the vertical axis. The largest neurons were at the L4 border, interspersed with rare and small granular-like cells. L4 was not totally incipient, but marked by small, granule-like cells diffused along the L3-L5 border, and a quite obvious white matter band separating them. The relatively thin L5 was composed of mostly large pyramidal cells, transitioning into L6 showing sparser and sparser cells, letting in bundles of myelin in, which did not go beyond L5.

**Fig. 18:**
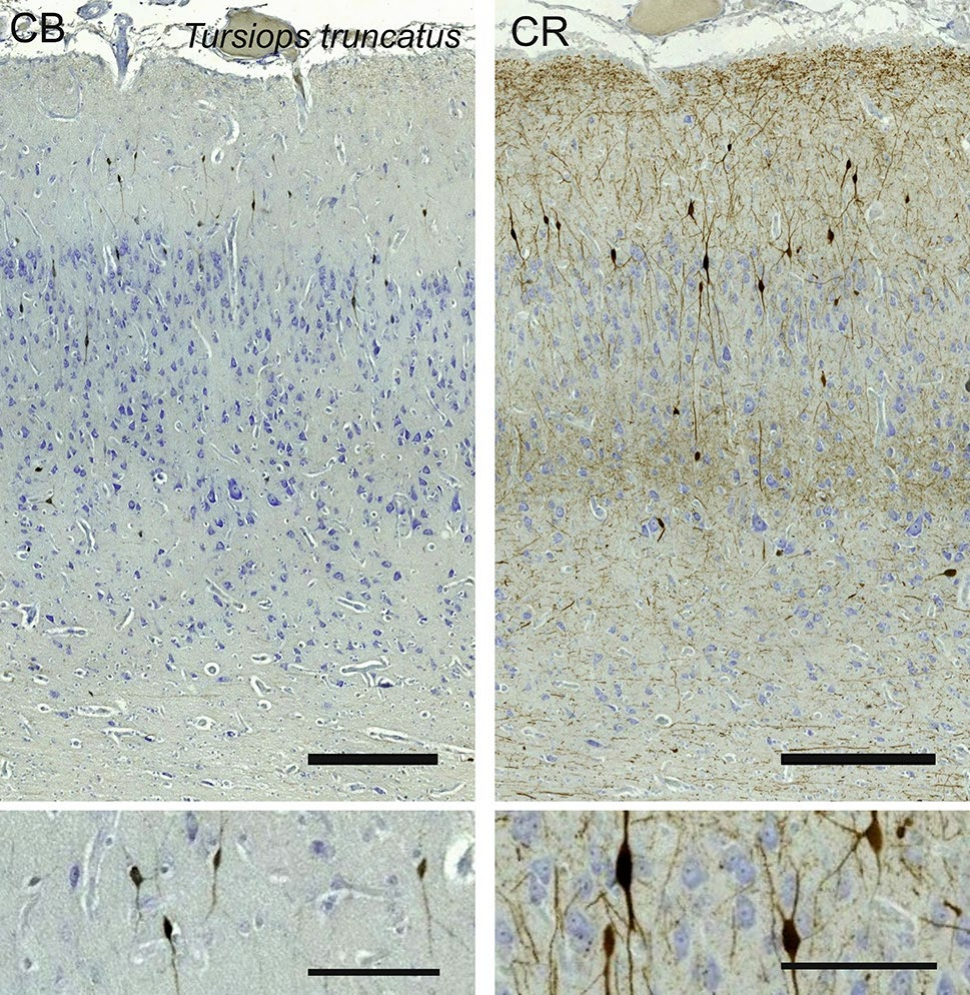
Microphotographs of CB-ir (left) and CR-ir (right) neurons in the primary visual cortex of the bottlenose dolphin (*Tursiops truncatus*), with magnified inserts below. Bars are 300 μm, 100 μm in inserts

CB-ir cells were almost all bipolar (Fig. 18), oriented vertically, and bound to L1-L3 with very few incursions into upper L5. CB-ir fibers were also found in the upper L1, near the pial surface. On the other hand, CR immunostaining revealed additionally a clear neuropil band at the L3/L5 border (Fig. 18). This CR-ir band of fibers protrudes in both layers. The cells are mostly bipolar found in L1 and L2, with somata sizes ranging from middle sized pyramidal cells to the smallest of neurons.

### Risso’s dolphin (*Grampus griseus*)

**Fig. 19:**
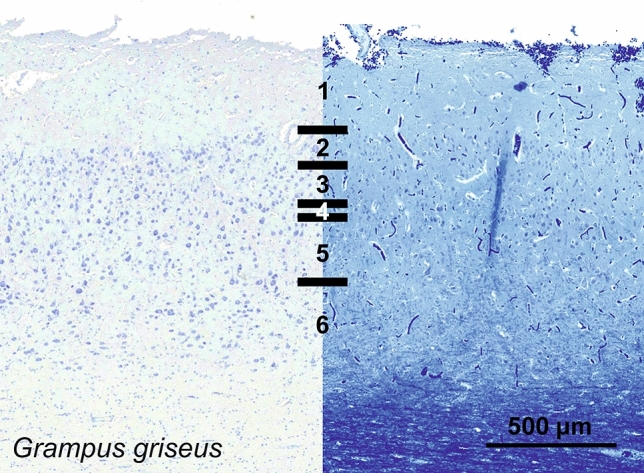
Photomicrograph of Nissl (left) and Kluver–Barrera (right) stain of the primary visual cortex of Risso’s dolphin (*Grampus griseus*). The images have been voluntarily slightly overexposed to enhance the structure visualization

In the visual cortex of *Grampus griseus*, the depth of the mantle was reduced to little over 1 mm (Fig. 19). L1 was relatively much thicker, together with L2. The general aspect of the cells was very homogeneous; in particular showing large L2 neurons characterized only by their packed quality. L3 and L5 were difficult to tell apart, with very rare granular-like cells dispatched therein. L3 contained relatively smaller cells than L5, and was seemingly denser, while L6 was interspersed by wide spaces of white matter. Myelin staining did not provide obvious fiber bundles, and little myelin beyond L6.

**Fig. 20:**
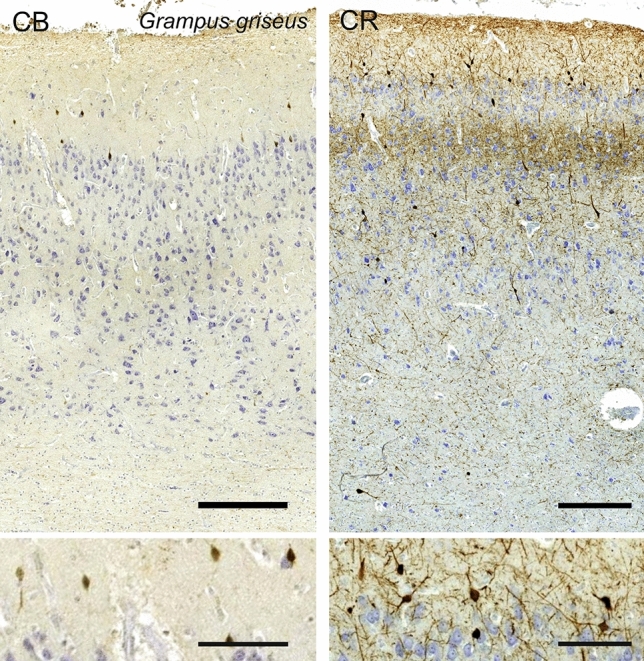
Microphotographs of CB-ir (left) and CR-ir (right) neurons in the primary visual cortex of Risso’s dolphin (*Grampus griseus*), with magnified inserts below. Bars are 300 μm, 100 μm in inserts

Immunoreactive neurons for CB were scarce and limited to lower L1 and L2, with a typical ovoid shape and their primary dendrites orientated vertically (Fig. 20). On the other hand, CR-ir neurons were rather extensively present in the cortex of Risso’s dolphin. Very dark interneurons were found from L1 to L6, most of which in lower L1 and L2, but also clearly present in L3, L5 and L6. The ovoid bipolar aspect of most of the interneurons present in L1 to L3 then changed to include multipolar cells. At the border between L3 and L5 was evidently seen a rather broad neuropil band, from mid L3 to right above the largest neurons of L5 (Fig. 20). This very dense entanglement of varicose was relatively devoid of CR-ir cells, and echoed the typical dense band also present in the upper molecular layer L1. Numerous varicosities were also seen crossing vertically from the many CR-ir cells in upper L2 to the CR-ir nerve fiber plexus in the marginal zone/L1.

### Long-finned pilot whale (*Globicephala melas*)

**Fig. 21:**
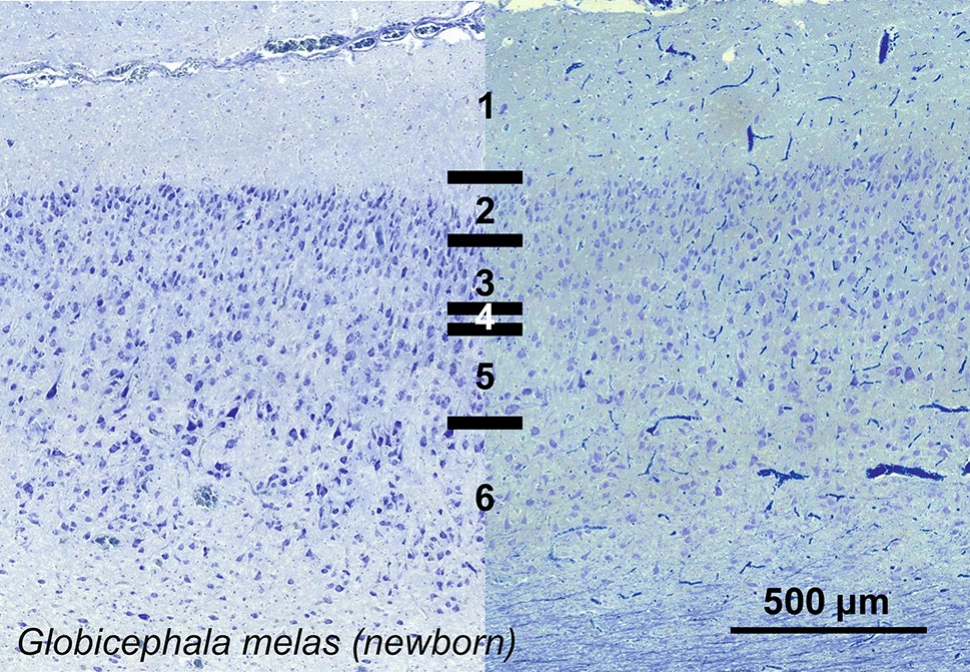
Photomicrograph of Nissl (left) and Kluver–Barrera (right) stain of the primary visual cortex of the long-finned pilot whale (*Globicephala melas*). The images have been voluntarily slightly overexposed to enhance the structure visualization

The V1 of *Globicephala melas* showed a rather dense neuronal population in a thin mantle depth. L1 was thicker than any other below layers, devoid of cells and bordered by a poorly differentiated L2 containing middle-sized neurons. Although several neurons appeared round, the morphology of the neurons varied, including some very discrete angular pyramidal cells, in L2, L3 and particularly in L5 (Fig. 21). A thin L4 led directly to a thicker L5 without any clear L4. Lower L5 and upper L6 neurons were pyramidal, well-marked with a dendritic cone orientated vertically, while L6 contained much smaller neurons with rounder shapes.

**Fig. 22:**
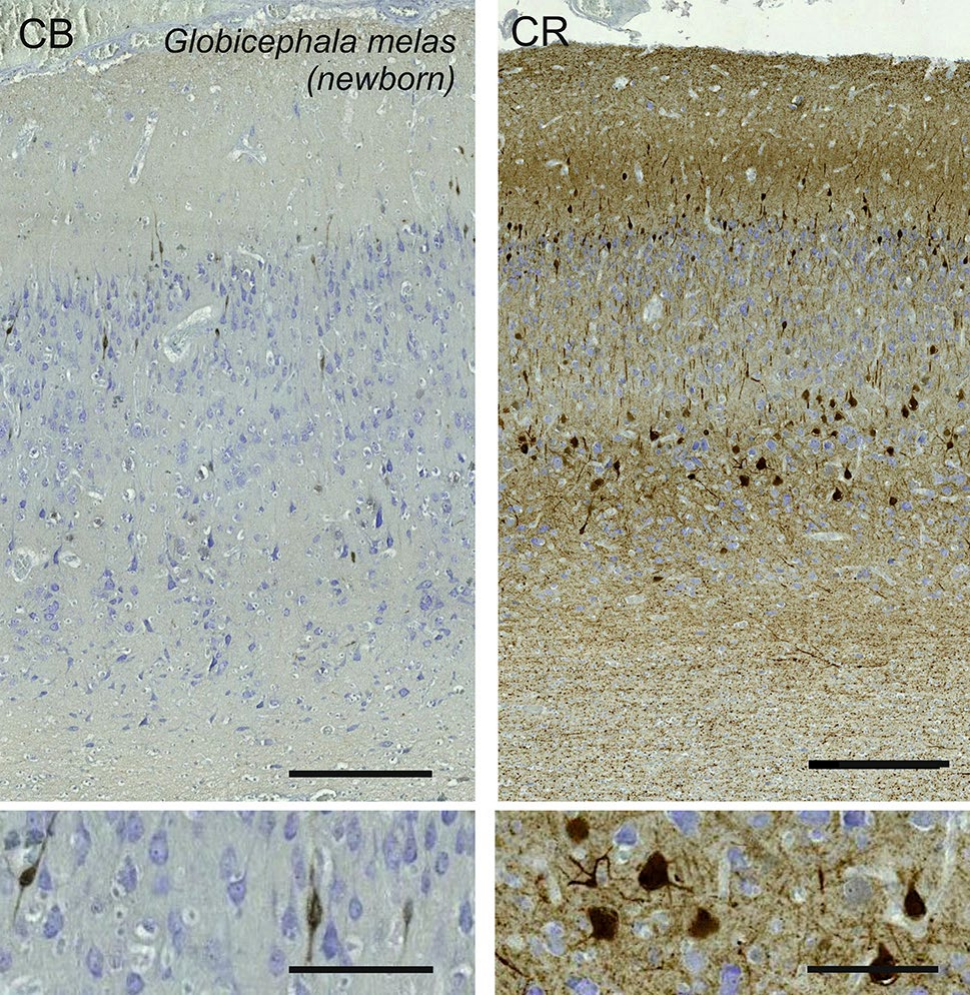
Microphotographs of CB-ir (left) and CR-ir (right) neurons in the primary visual cortex of the long-finned pilot whale (*Globicephala melas*), with magnified inserts below. Bars are 300 μm, 100 μm in inserts

Bitufted CB-ir neurons were found mostly limited to lower L1 and L2, with some positive nerve fibers present in the marginal zone/L1 (Fig. 22). Calretinin was found in more neurons, with fusiform and multipolar types ranging from L1 to L6 (Fig. 22). The amount of CR-ir neuropil was diffuse throughout the cortex, but formed two clear bands, one in L1, and the other in L5, both relatively close to cell clusters.

### Cuvier’s beaked whale (*Ziphius cavirostris*)

**Fig. 23:**
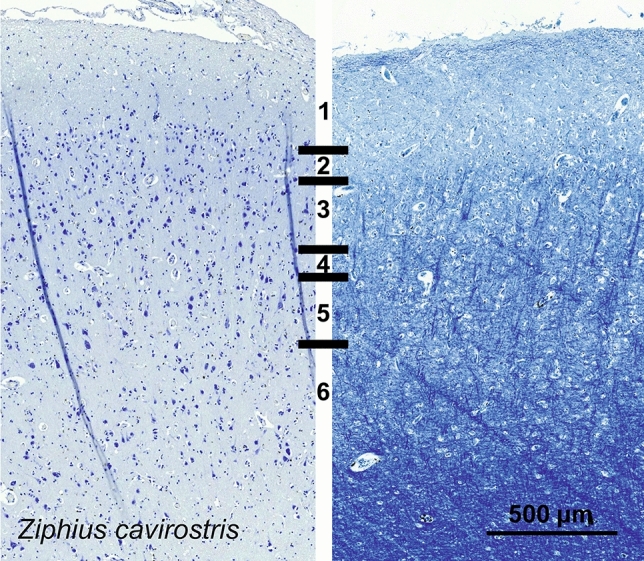
Photomicrograph of Nissl (left) and Kluver–Barrera (right) stain of the primary visual cortex of Cuvier’s beaked whale (*Ziphius cavirostris*). The images have been voluntarily slightly overexposed to enhance the structure visualization

In the V1 of *Ziphius cavirostris*, L1 was slightly thinner than in the bottlenose dolphin. Neurons in L2 were also smaller. The transition to L3 was diffuse, and L4 was only discernible via very small rounder cells disseminated in the lower L3 and upper L5 and a gap of neuropil (Fig. 23). L5 was relatively present and layer 6 markedly sparser in cells. The myelin stain showed numerous bundles of myelinated fibers oriented vertically, and no obvious myelin band at the margin between layer 3 and 5.

**Fig. 24:**
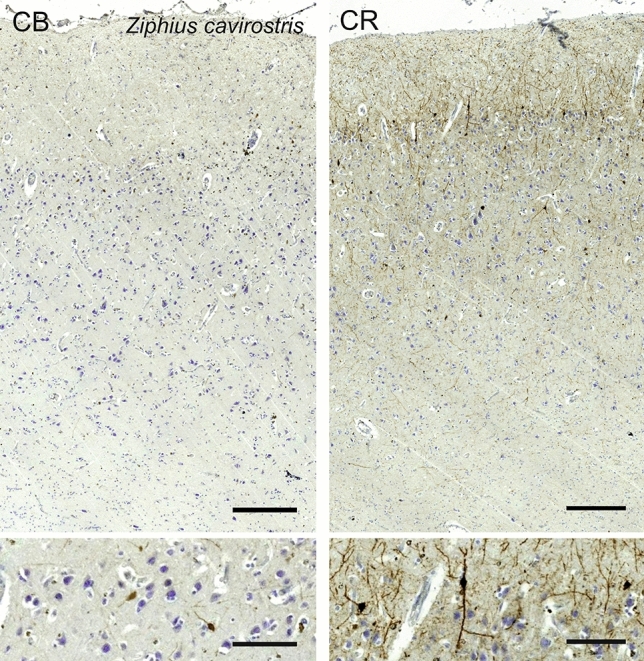
Microphotographs of CB-ir (left) and CR-ir (right) neurons in the primary visual cortex of Cuvier’s beaked whale (*Ziphius cavirostris*), with magnified inserts below. Bars are 300 μm, 100 μm in inserts

Few CB-ir interneurons were seen from lower L1 down to L3, fusiform with one or two primary dendrites apparent (Fig. 24). Neuropil marking was mostly limited to the upper L1 and around the CB-ir themselves. CR-ir interneurons were mostly present in the vicinity of L2, with some somata immunoreactive in L3 and even L5. The majority was composed of bipolar cells with the primary dendrites emerging from the poles (Fig. 24). Some rare neurons presented a more multipolar and angular appearance, particularly in L5. The neuropil distribution was evident in L1 and L2, stemming from the L2 neurons, but some dendrites, apparently in bundles descended vertically in to the L5–L6 border.

### Sperm whale (*Physeter macrocephalus*)

**Fig. 25:**
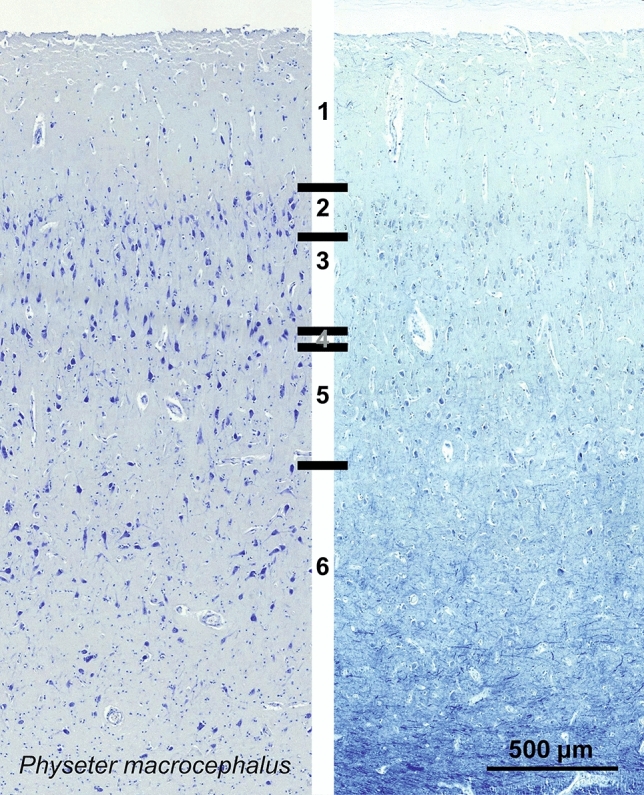
Photomicrograph of Nissl (left) and Kluver–Barrera (right) stain of the primary visual cortex of the sperm whale (*Physeter macrocephalus*). The images have been voluntarily slightly overexposed to enhance the structure visualization

**Fig. 26:**
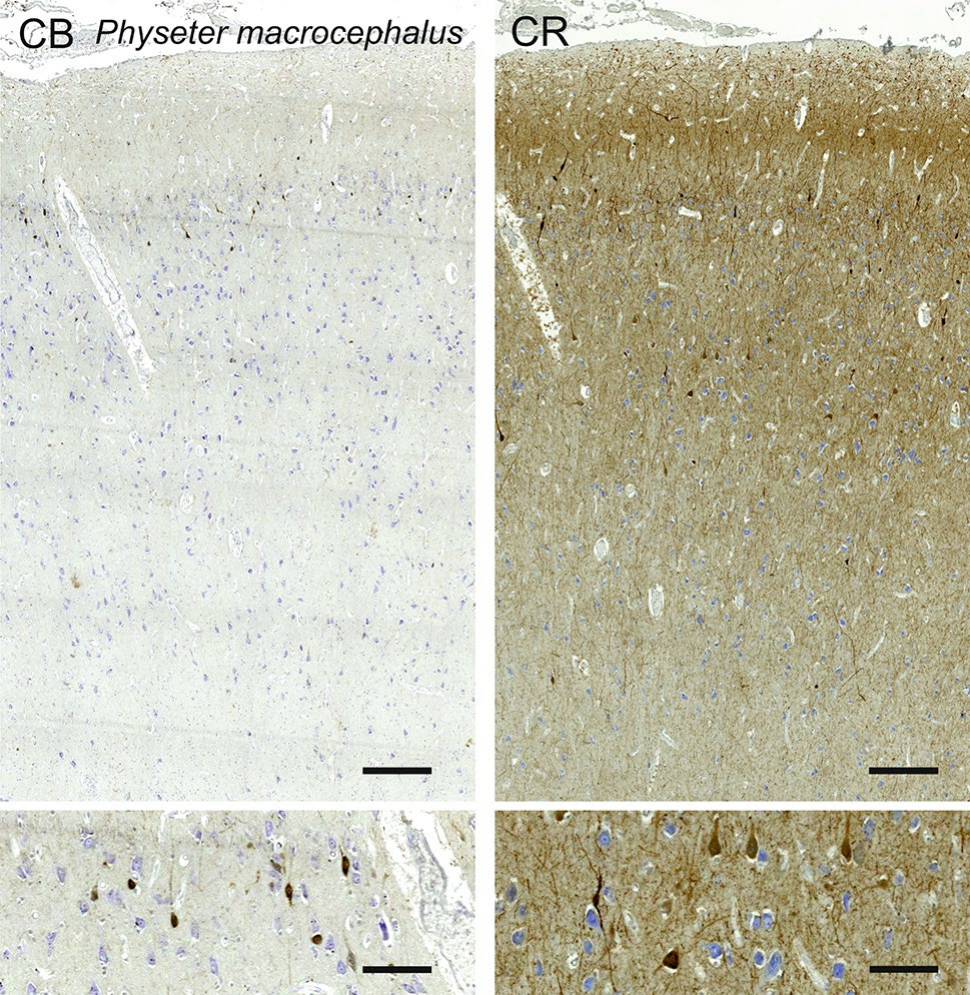
Microphotographs of CB-ir (left) and CR-ir (right) neurons in the primary visual cortex of the sperm whale (*Physeter macrocephalus*), with magnified inserts below. Bars are 300 μm, 100 μm in inserts

The primary visual area of *Physeter macrocephalus* displayed a relatively sparse, but thick cortex (Fig. 25). Throughout the cortical thickness, neurons ranged from small multipolar cells to large pyramidal cells orientated vertically. The limit between L2 and L3 was blurred by the lack of clear neuron morphology difference. Rare minute granule-like neurons defined a thin L4 separating the large pyramidal cells in L3 and L5, with a yet lower density in L5. L6 was sparser still, with several multipolar cells. Myelin was not obviously organized in vertical bundles, rather horizontal fibers in L6 and to a lesser extent in L5. Much like in other cetaceans,

CB-ir neurons were rather limited to L1 and L2, bordering with L3 (Fig. 26). Fusiform neurons oriented vertically composed the vast majority of the cell types. Neurons immunoreactive to CR were distributed along the whole cortical thickness, mostly comprising fusiform (bitufted) cells with long dendrites protruding from both poles (Fig. 26). Some of the lower CR-ir cells in L3 to L5 were also appearing multipolar, but with their dendrites orientating mostly vertically. Rich varicosed protrusions were forming plexuses in L1 and L5, the latter more easily seen macroscopically.

### Fin whale (*Balaenoptera physalus*)

**Fig. 27:**
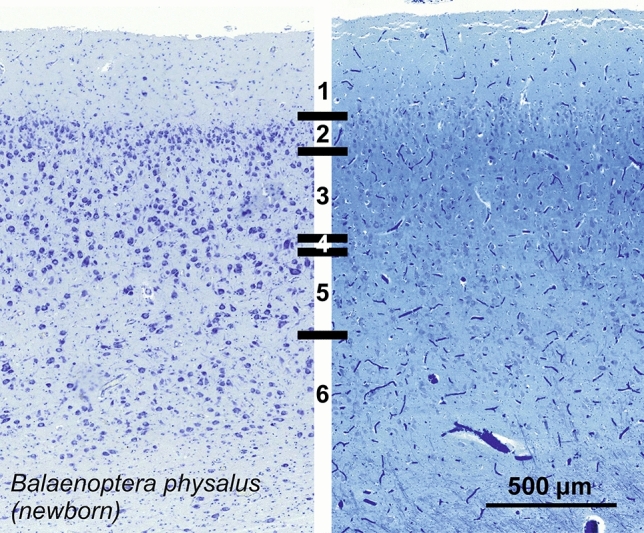
Photomicrograph of Nissl (left) and Kluver–Barrera (right) stain of the primary visual cortex of the fin whale (*Balaenoptera physalus*). The images have been voluntarily slightly overexposed to enhance the structure visualization

The visual cortex of *Balaenoptera physalus*, albeit relatively thin, displayed discrete patterns, with an average cetacean L1, but a dense and granular-like L2 composed of small round neurons (Fig. 27). The transition into L3 was progressive, characterized by larger neurons taking a more pyramidal appearance. At the margin between L3 and L5, Neurons were clearly large, and attempts to find a layer 4 was limited to a slim neuropil band and rare granule-like cells. L5 was marked typically large pyramidal neurons with varying orientations in a sparser layout than L3. Finally, L6 was distinctively devoid of the large cells of L5 and populated by sparse smaller ovoid neurons, orientated rather horizontally, reminiscent of fusiform cells in other L6 of mammals. Myelin physical staining showed only a darker aspect in layers 2 and 3.

**Fig. 28:**
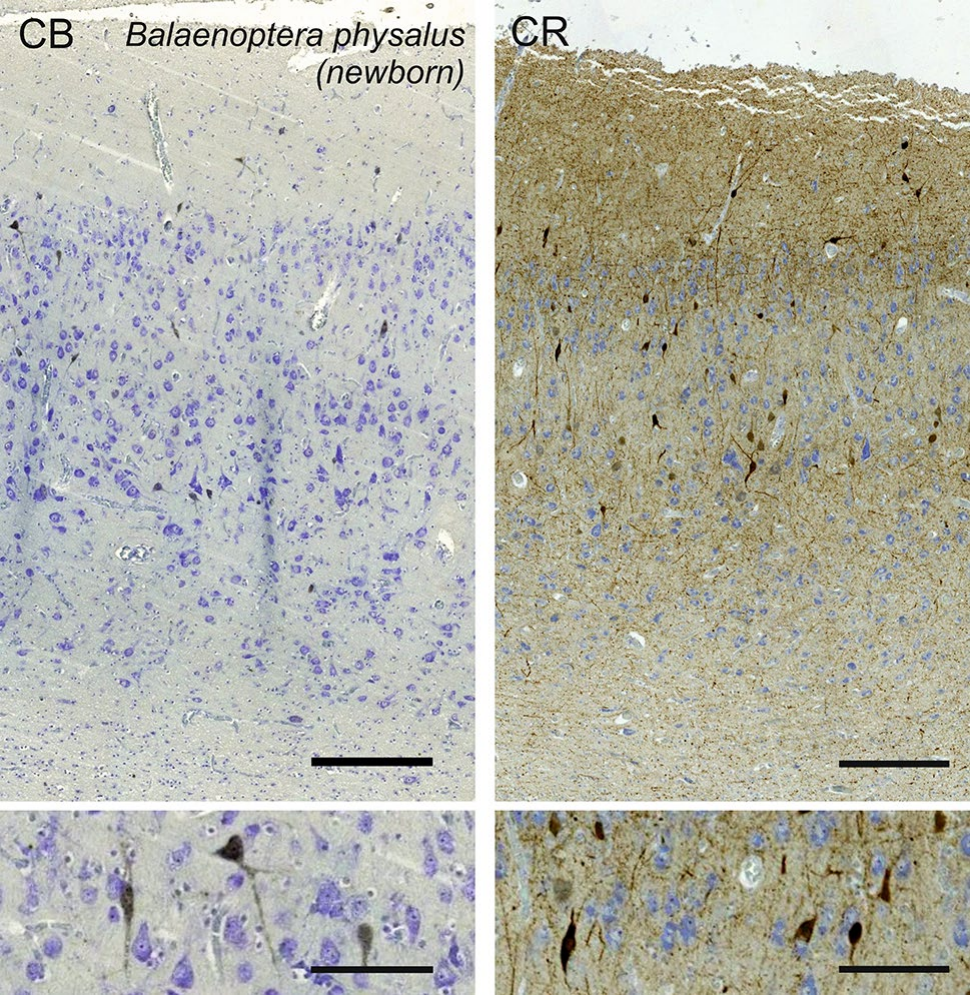
Microphotographs of CB-ir (left) and CR-ir (right) neurons in the primary visual cortex of the fin whale (*Balaenoptera physalus*), with magnified inserts below. Bars are 300 μm, 100 μm in inserts

Immunocytochemistry for calbindin yielded a limited reactivity in the fin whale, with very few CB-ir neurons weakly marked in L2-L5 (Fig. 28). The reactive cells were bipolar or multipolar, with short protrusions marked. The staining for CR yielded more reactivity, with more mostly bipolar neurons ranging from L1 to L5 (Fig. 28). Immunoreactive varicosities were spotted throughout L1 and L2, and in the upper L5.

### Statistical analysis

Statistical results are presented in full in the Supplements. The most important results are mentioned below.

There was no striking result coming from grouping specimen by water *vs.* terrestrial or by their diet (carnivore, omnivore, herbivore).

**Fig. 29:**
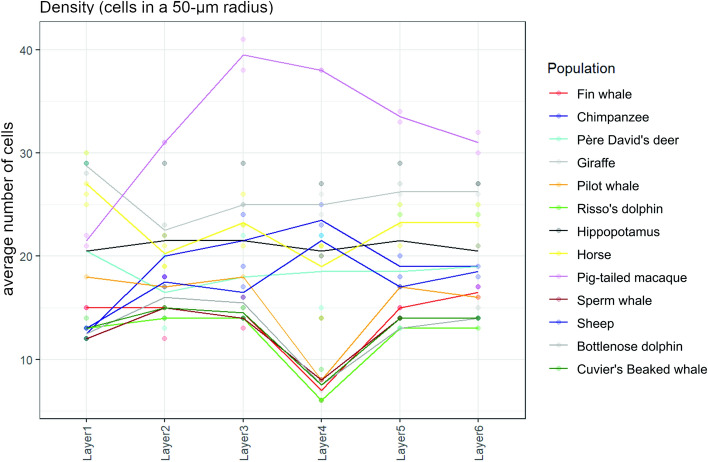
Graphical representation of the surface density measurements made by layer for each species in our sample. The unit of density is the median number of cells found in a 50-micron radius around a given cell. The dots above and below each line represent the variability (scatter) for each layer

The statistical analysis organized in groups per species identified trends without establishing significance. The macaque had the highest density in cells in absolute, being the highest in L3 and L4 (Fig. 29). Waterborne species gathered on the lower end for all layers, while terrestrial ungulates were intermediately positioned.

**Fig. 30:**
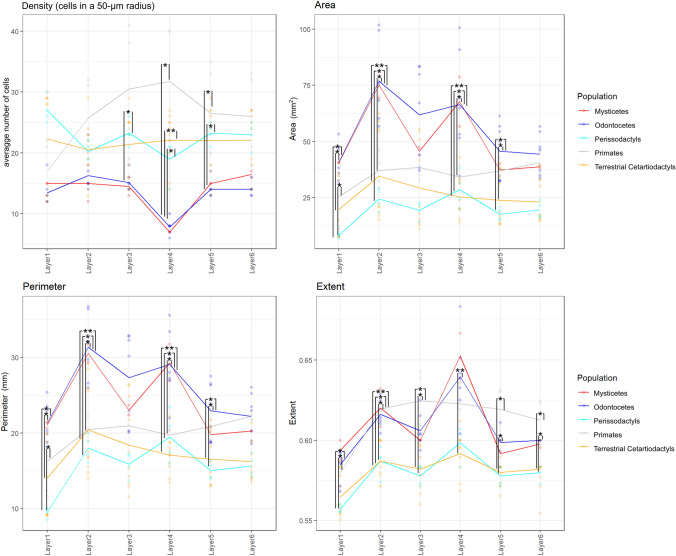
Graphical linear distribution of selected parameters of the density (median number of cells found in a 50-micron radius around a given cell, top left panel), size (area and perimeter in microns, top right and bottom left panels) and shape (extent = ratio of pixels in the region to pixels in the total bounding box, returned as a scalar. Computed as the Area divided by the area of the bounding box) (bottom right panel) domains by layer for our samples regrouped by taxonomy. Significant differences are marked by asterisks (*for *p* ≤ 0.05; **for *p* ≤ 0.01)

In the analysis where specimens were grouped by *phylogenetic group*, density was clearly the highest in primates, thrice that of cetaceans, which were quite similar together, and an intermediate position was found for ungulates, including perissodactyls and terrestrial Cetartiodactyls. In particular, layer 4 in primates showed the highest concentration of cells (Fig. 30 top left), and cetaceans (mysticetes and odontocetes) had the lowest concentration (*p* < 0.05 against all other groups). The dimensions of the neurons were notably larger in cetaceans than in the other taxa (odontocetes and mysticetes *p* < 0.05 in L2 and L4). In both L2 and L3, especially in cetaceans, the distribution of data was much wider, with smaller and larger cells (Fig. 30 top right). The variation in neuron size (Area, Perimeter, for the others see Supplements) was much more evident in cetaceans and ungulates than for primates, which deviated little across layers compared to the other taxa (Fig. 30 top right and bottom left). Throughout L1 to L6, the perissodactyls and terrestrial Cetartiodactyls were homogeneous, with no specific differences. The analysis of neuron shape showed that L1, L5 and L6 had the most regular neurons, with L1 most so (Fig. 30 bottom right). The irregularity on the contrary of L2 and L4 was most evident (in particular for cetaceans against ungulates in L4 *p* < 0.05; Fig. 30 bottom right). Interestingly, the values for primates did not show such variations, with L4 being relatively homogeneous. InvAR also suggested a relative irregularity across layers in all taxa but primates, and a comparatively large variation within each layer, in particular for L2, L3 and L4.

### Laterality and binocularity

**Fig. 31:**
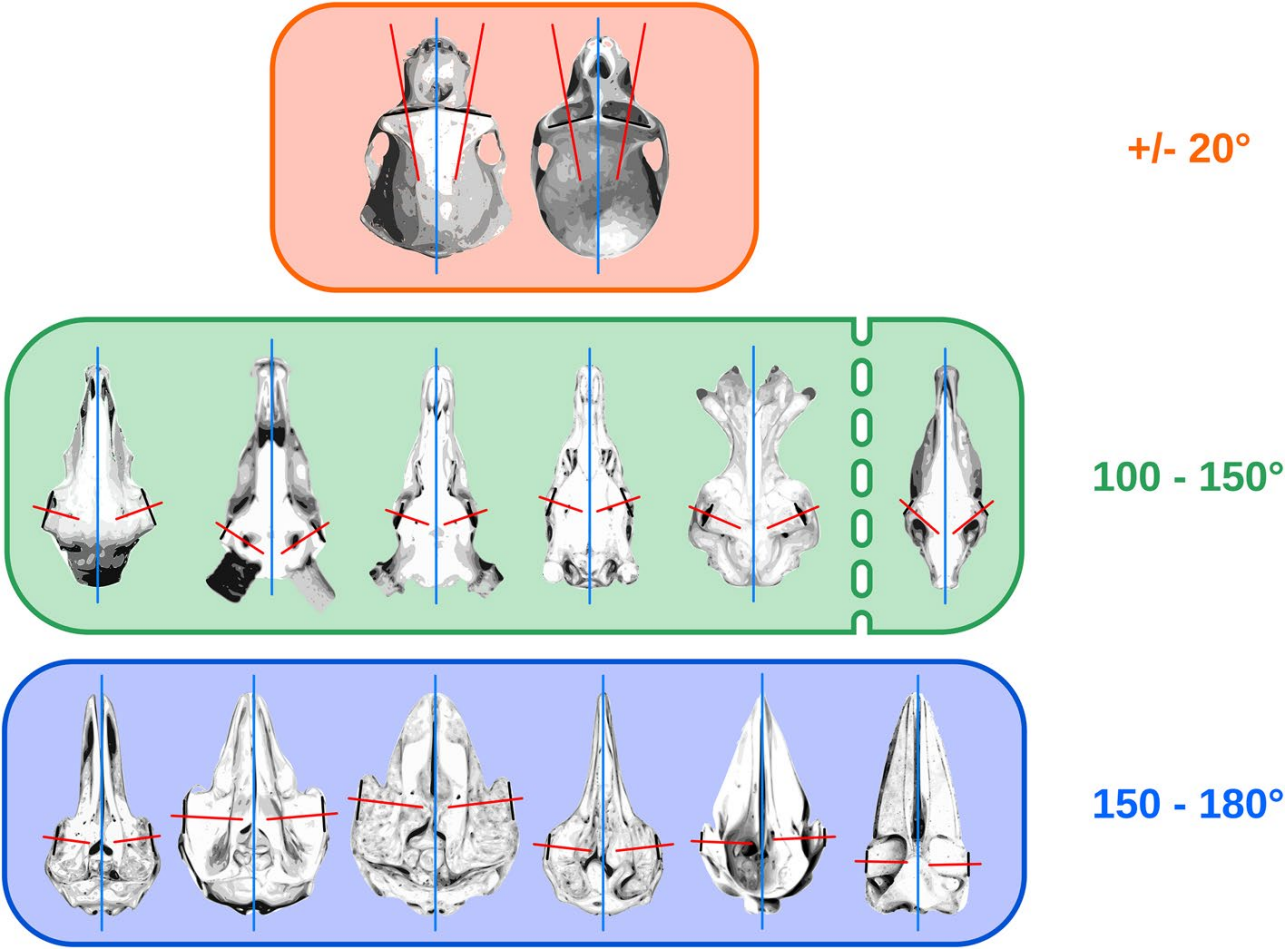
Illustration of the eye position and orientation based off the orbital plane of the skulls from animals in the present work. This extrapolation is not accurate enough to predict correctly eye field of vision (Duke Elder, 1961); however, it remains helpful to visualize gross differences in the position resulting in the grouping in three categories that we named frontal-eyed (represented by primates), wide field (terrestrial Cetartiodactyls) and lateral-eyed (cetaceans)

Considering roughly each of the species skulls, eye position and orbital planes, we categorized the animals into three groups, the first with the orbital planes forming and angle around 20° (gamma ± 10°), comprising the primates, the second with orbits axes forming an angle of 100° to 150° (gamma between 50° and 75°), encompassing all the Artiodactyls, and a third group, cetaceans, with their orbital planes angled from 150° to 180° (gamma between 75° and 90°) (illustrated in Fig. 31).

**Fig. 32:**
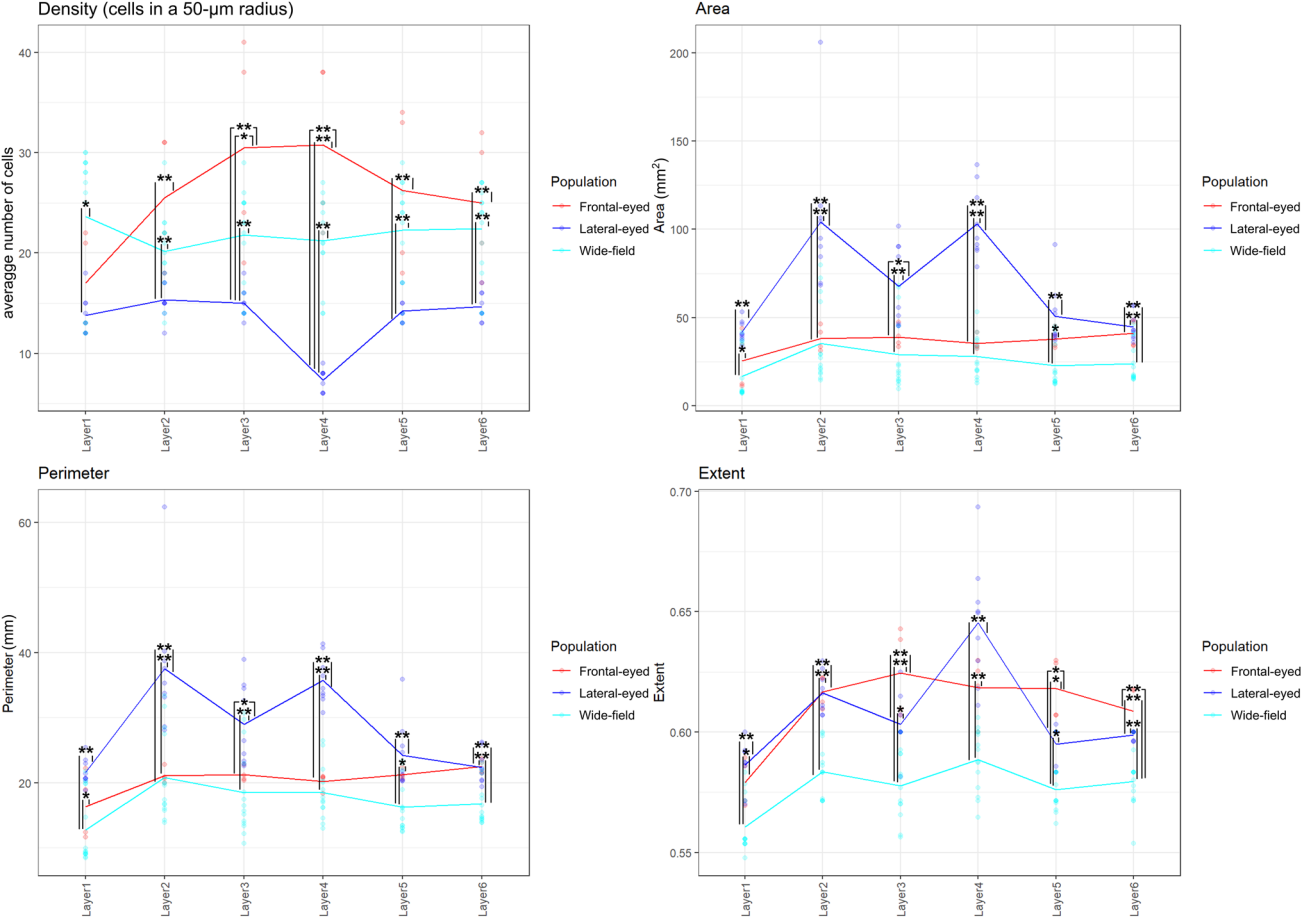
Graphical linear distribution of selected parameters of the density (median number of cells found in a 50-micron radius around a given cell, top left panel), size (area and perimeter in microns, top right and bottom left panels) and shape (extent = ratio of pixels in the region to pixels in the total bounding box, returned as a scalar. Computed as the Area divided by the area of the bounding box) (bottom right panel) domains by layer for our samples regrouped by eye position. Significant differences are marked by asterisks (*for *p* ≤ 0.05; **for *p* ≤ 0.01)

The analysis of specimens grouped per *eye position* (frontal-eyed, wide-field and lateral-eyed) demonstrated a disparity between groups in density, shape and size. In particular, lateral-eyed subject showed significantly lower density (*p* < 0.01 in L2–L6) (Fig. 32 top left), a significantly different shape (*p* < 0.05 on almost all layers), and larger dimensions (*p* < 0.05 in L1, L2 and L4) in upper layers than frontal-eyed and wide-field groups. Frontal-eyed animals (chimpanzee and macaque) had the most regular (*p* < 0.05 in L3–L6) and most dense (*p* < 0.05 in L3 and L4) neurons but not the smallest, which were in the wide-field group.

## Discussion

The Order Cetartiodactyla includes two clades, the terrestrial Artiodactyla (even-toed mammals) and the marine Cetacea (dolphins and whales). The fusion in a single order (or SuperOrder) derives by molecular evidence pointing to a common artiodactyl precursor from which marine cetaceans differentiated in early Eocene (> 50 million years ago). The resemblance between terrestrial artiodactyls and their marine cousin cetaceans is difficult to grasp immediately, but the position of the eyes, quite lateral in the head, is evident in all species. This latter characteristic may be related to the necessity to scan the horizon for the terrestrial herbivores, but the same does not necessarily apply to cetaceans that include classic predator species (toothed whales) that rely on echolocation for hunting, and krill filterers (whalebone whales). The present study started from simple questions: does the visual cortex of Cetartiodactyls follow a common evolutionary Bauplan and share organizational features? Are there differences between terrestrial and marine species of the same Order, or even among species that share the same environment but belong to different families? To better understand the evolution of the visual cortex of Cetartiodactyls we studied several members of the Order. We also compared the results obtained with data from the closely related herbivore perissodactyls, and with findings in primates, whose visual cortex is generally considered to be the most complex because of the advanced extent of the binocular field and stereoscopy.

In a last few years, our group analyzed the brains of large ungulates, including the horse (Cozzi et al. 2014a, b, 2017b), the bovine (Peruffo and Cozzi 2014; Ballarin et al. 2016; Graïc et al. 2018; Corain et al. 2020); the pig (Minervini et al. 2016); the sheep (Peruffo et al. 2019) and the giraffe (Graïc et al. 2017). The cytoarchitectonics of these mammals indicates a substantial difference from primates and rodents, with a consistent reduction of L4 and different targets of thalamic afferent within the cortical column (for details and reference see Cozzi et al. 2017b; Peruffo et al. 2019). The cytoarchitectonics of the cetacean brain has been the subject of numerous studies (Breathnach 1960; Morgane et al. 1986; Ridgway 1990; Glezer et al. 1992b, 1995; Poth et al. 2005; Kern et al. 2011; Butti et al. 2011, 2014a,b; van Kann et al. 2017; for general reference see Cozzi et al. 2017a; Huggenberger et al. 2019). Specifically, the cetacean visual cortex has been thoroughly studied by relatively few authors (Kesarev and Malofeeva 1977; Morgane et al. 1985, 1988, 1990; Oelschläger et al. 1987; Garey et al. 1985; Garey and Leuba 1986; Garey and Revishchin 1986; Garey et al. 1989; Glezer et al 1992a, 1993; Manger et al 1998; Graïc et al. 2021), sometimes with a broad comparative scope (Glezer et al. 1998; Hof et al. 2000; Raghanti et al. 2014; Cozzi et al. 2017a; van Kann et al. 2017). In general, the organization of the cetacean cerebral cortex differs from that of terrestrial mammals, with predominance of phylogenetically older L1 and L6, a poorly differentiated population of relatively large neurons, and consequently a low degree of granularity (Morgane et al. 1990). Furthermore, direct comparison of absolute thickness, lamination, myelination and cytoarchitecture indicates that there is no unique cetacean type, as found in other taxa (Krubitzer et al. 2011) but different variations, further emphasized by immunocytochemical results. Our data confirm that specific characteristics of the cetacean cortex are more evident in the visual cortex (Figs. 30, 31), as already reported (Morgane et al. 1988). In particular, the size of cetacean cells in “granular” layers (L2 and L4) were notably larger than that of ungulates, either terrestrial cetartiodactyl or perissodactyl, and of primates. Again, this tends to confirm a certain lack of granular cells and lamination. However, interestingly, odontocetes and mysticetes had the widest variation of size across layers, with a wide variability (Fig. 30). Granted that the low number of specimens might explain part of it, this nevertheless suggests the existence of different neuron sizes within each layer, which seems to be confirmed by CBP immunostaining.

### Lamination

The lamination and cytoarchitectonics of the visual cortices of the two primates that we studied fully correspond to what described in classic texts and references (see Blümcke et al. 1990; Morrison et al. 1998). Calculation of cortical thickness in the chimpanzee and macaque suffers from the limitations of our sample size and age (Natu et al. 2019), but the results for the macaque agree with values reported by others for the primary visual cortex (1.5 to 1.6 mm thick; Peters and Sethares 1991).

The visual cortex of most Cetartiodactyls and of the horse was poorly laminated if compared to those of the macaque and chimpanzee. Interestingly, statistical analysis showed a relatively high homogeneity between even-toed and one-toed ungulates, correlated with a closer phylogenetic position compared to the other taxa tested (Fig. 30).

Our results in the sheep partially agreed with what was reported by Rose (1942). The cortex is thin and relatively rich in cells. L2 and L3 are difficult to separate, L4 is discreetly wide and can sometimes be separated into L4a containing feebly staining, small granular cells, and L4b with darker more pointed cells. L5 is quite thin and contains few large neurons, and yet fewer much larger cells. L6 is wider than L5 and quite dense. Its cells are arranged in rows and generally smaller than those of L5.

In the other species, although inherently variable with cortical region and circumvolution positioning, L1 was distinctively large compared to primates, as previously reported. L1 was particularly large especially in the sperm whale, giraffe and Risso’s dolphin.

In all ungulates, L2 was also usually larger and less dense, as previously described (Morgane et al 1985). There are analogies with the visual cortex of bats, including the presence of extraverted neurons (Sanides and Sanides 1972). The large array of sizes present throughout L2-L5 in cetaceans (Fig. 30) suggests that the layered cortical pattern is more diffuse, and that granular, smaller neurons, including interneurons, are dispersed among inner and outer pyramidal layers.

The large granular lamination of L2 and L4, considered characteristic of sensory cortex, are reduced in all ungulates, but became evanescent in cetaceans, although previously signaled in V1 (Morgane et al. 1988; Supin et al. 2001; Graïc et al. 2020). The density results reflect this fact, as L4 is extremely low in cetaceans, this being due to the sparse distribution of few recognizable cells in the area, hardly forming an actual layer.

In most cases, identification of L4 at the L3–L5 border was problematic. The presence of granule cells by Nissl staining and/or the appearance of a layer of myelin in Klüver-Barrera staining are the simplest form of inner granular layer sign. In some species, a proper L4 was identifiable, as in the giraffe, where the presence of a L4A and C was hinted by smaller granule-like neurons with a cell-poor thin stripe in between (Fig. 13) as previously described in Jacobs et al. (2014). In the horse, L4 was equally shrunk to a minimum, coherent with previous publications (Cozzi et al 2017b). The difficulty for the community to agree on a limit between L3 and L4 exists also in primates (Peters 1994; Balaram et al. 2014); nevertheless, the limit between almost all other layers is blurred in most terrestrial Cetartiodactyls and particularly in cetaceans.

According to (Morgane et al. 1988), the primary visual cortex of the striped dolphin is heterolaminar and contains a weak (“incipient”) L4 with distinct granules, which has recently been found in the long-finned pilot whale (Graïc et al. 2021), and differentiate V1 from V2. In the same study, Morgane and colleagues (1988) found by Golgi staining that L2 contained extraverted neurons with widespread spinous apical dendrites directed towards L1. The presence, distribution, and morphology of these features in both these species was hinted in our work in the Risso’s dolphin, another *Delphinidae*, but not as clearly in the Cuvier’s beaked whale, sperm whale or fin whale.

As can be seen in Fig. 1, the distinct line of Gennari progressively fades from primates to cetaceans, although it can still be faintly seen. Our results suggest the presence of a circuitry at least partially different from that of primates. Yet, a different circuitry does not imply lesser performance, as the use of visual cues has been reported repeatedly even in several marine Cetartiodactyls (von Fersen et al. 2000; Reiss and Marino 2001; Karenina et al. 2010; Mitchell et al. 2013; Tomonaga et al. 2014; Knolle et al. 2017).

The organization of the *fasciculi* is clear in the myelin stains. However, the columnar arrangement usually well seen in primates (Braak 1976; Figs. 3, 5), is not obvious in ungulates. Rather, myelin histological stains show some bundles arising from the white matter and expanding through the cortical grey. This organization is relatively present in both terrestrial and marine Cetartiodactyls, and in Perissodactyls. There are variations among the species with a pattern that suggests a progressive evolutionary shift in cortical organization. In fact, we noted a more definite aspect, and consequently clearer identification of the myelin bundles, in the sheep and horse. The deep diving sperm whale is at the opposite end, with no clear bundles despite a thick, neuropil-rich cortex (Fig. 25).

### Immunohistochemistry of the calcium binding proteins

The existence of GABA-ergic cells in V1 has been extensively described in primates (Peters 1994; Jones et al. 1994; Morrison et al. 1998; Ulfig 2002), and is part of the visual area circuitry both for parallel processing and signal enhancement. GABA-ergic neurons are in large majority interneurons (DeFelipe et al. 2013), which are critical for signal modulation throughout sensitive pathways, naturally including the cortex (Fino et al. 2013). More importantly, evidence showed that inhibitory neurons varied in relative proportions among mammalian groups and relative to brain size (Hof et al. 1999; Sherwood et al. 2007, 2009). There is also evidence that those interneurons are not exclusively local, but reach the white matter (Micheva et al. 2016), and have been proven to connect relatively distant areas (Melzner et al. 2012). Calcium-binding proteins (CB, CR and PV) which have been shown to identify almost exclusively non-overlapping populations of GABAergic neurons (Andressen et al. 1993; Glezer et al. 1993; DeFelipe 1997).

### CBPs as GABA markers in terrestrial and semi-aquatic species

Our data substantially confirm previous histological studies on the lamination and organization of the giraffe, including the presence of neuronal morphology typical of Eutheria (Jacobs et al. 2014). Immunohistochemical investigation indicates the existence of a marked neuropil band in L4, as demonstrated both by PV and CR. On the contrary, CB-ir was much less useful to see the structure of the cortical layers than the former two. The CR-ir neurons in our hippopotamus V1 closely resemble those already described in the pygmy hippopotamus (Butti et al. 2014a). The difference in complexity between the giraffe and hippopotamus V1 follows similar findings made in their respective retinas (Coimbra et al. 2013, 2017).

Alternatively, CR clearly marked neuropil in L1 of the sheep, deer, giraffe, hippopotamus, and horse. Somata were most numerous in the giraffe, in which CR-ir neurons were dispersed also in L2 and L3 (Fig. 14).

In the macaque and chimpanzee, PV-ir neurons identified a band corresponding to L4C, known from the literature to receive both magnocellular and parvocellular inputs from the lateral geniculate nucleus (LGN) (Sincich and Horton 2005; Fitzpatrick et al. 1987). Several PV-ir neurons marked a distinct neuropil band in the sheep and in Pere David’s deer. In the giraffe the latter band was doubled with somata visible throughout and in between the two bands. In the horse the said band did not split in two, but seemed to expand towards L3. The distribution of PV-ir neurons was much more diffuse in the hippopotamus, a semi-aquatic species.

### CBPs as GABA markers in cetaceans

We are aware that CR is a powerful tool to detect GABAergic neurons, especially in the central nervous system (Glezer et al. 1992b). Former comparative studies in Cetartiodactyls and primates noted that CR-containing neurons were much more abundant and brightly stained than CB-ir ones (Hof et al. 2000; Graïc et al. 2021), as we also report. However, in our experimental series most CR cells were distributed in the upper layers L1-L2, but also in L3 and L5. Additional to the fusiform neurons described by Glezer and coworkers (Glezer et al. 1992a, b), we identified multipolar cells (Fig. 20). Furthermore, in our specimens the neuropil ramified into bands impossible to miss. Interestingly, the visual cortex of the pilot whale (Fig. 22) contained a higher number of cells compared to other cetaceans, with an exceptional amount of neuropil. We were not able to identify PV-ir neurons in the visual cortex of cetaceans, as already previously noted in the bottlenose dolphin (Glezer et al. 1998). Interestingly, former studies of our group failed to identify PV-ir neurons in the claustrum of the bottlenose dolphin (Cozzi et al. 2014a, b). We were not able to identify PV-ir neurons in the visual cortex of cetaceans, as already previously noted in the bottlenose dolphin (Glezer et al. 1998). Interestingly, former studies of our group failed to identify PV-ir neurons in the claustrum of the bottlenose dolphin (Cozzi et al. 2014a, b), although some other groups reported PV staining in subcortical areas of other cetaceans (Dell et al. 2016a, b). We also note that the potential issue of a clear PV staining in the cetacean cortex might raise questions as to the evolution of the CBPs in these mammals. In some cases, such as the immunostaining in the Cuvier’s beaked whale and the CB staining in the fin whale, there is the possibility of some influence of the time of fixation after death, always potentially difficult to determine in stranded wildlife.

### Significance of CBPs results

Comparison of immunohistochemical results with Nissl images strongly suggests that the cetacean visual cortex, composed of large pyramidal-like cells, is indeed furtherly subdivided in additional functional layers (Graïc et al. 2021). These, in turn, suggest potentially different connection patterns within the column and towards other areas. Species differences in the pattern of CBP distribution have been reported and discussed in the literature (Glezer et al. 1993; Sherwood et al. 2007; van Kann 2017). However, here we emphasize that the changes that we observed are unexpected and do not fully match the current view on the cortical column of marine Cetartiodactyls in the different species. The sperm whale, e.g., displayed two dense neuropil bands clearly marked, which do not correspond to the canonical view either of the mammalian visual cortex or even to the commonly accepted pattern for the cetacean cortex (Fig. 26). Conversely, the patterns observed in the fin whale, the only baleen whale in our series, were relatively like the more classic cetacean model, and similar to delphinids in general.

In our series, Nissl stain failed to identify what we would *conventionally* define a clear L4 layer in marine Cetartiodactyls, confirming what already reported by former studies. However, immunostaining did reveal a *diffuse* band of CR-ir neurons, that, although not identifiable in a separate layer, may indeed act as a *functional* L4, as also described in other mammals, (Fig. 18). The potential evolutionary significance of the prevalence and distribution of CR-ir neurons is worth noting, but requires further studies. It is generally assumed that thalamic afferents reach L4 and L3c (Sanides and Hoffmann 1969; Sincich and Horton 2005). The presence in cetaceans of GABA-ergic interneurons in the upper layers (L1 to upper L3) may point out that inputs reach the cortex mainly via L1 instead of L4, as in rodents, primates, and carnivores. However, the presence of a strong neuropil signal at the border between L3 and L5 implies synapsing either to efferent neurons in lower layers, or potentially to the other cortical afferents ascending from white matter. Moreover, specific cases such as the sperm whale might even support the coexistence of both routes. The neuropil band in L3–L5 seen with CR and the dense neuronal fiber plexus in marginal zone/L1 seen in the Risso’s dolphin (Fig. 20) suggest that the cetacean cortex is far more complex than that of the hedgehog model (Morgane et al. 1990). Thus, thalamic inputs may not be limited to L1, but are probably dual, as found in other mammals, with matrix (M) thalamic inputs arriving via L1 and core thalamic inputs passing through L4 (Jones 1998; Rubio-Garrido et al. 2009). This in turn would require the existence of a *functional* L4, albeit the neurons endowed with this role are diffuse in the neighboring L3 and L5.

## Laterality

Stereopsis is a characteristic of several vertebrates and is present also in species whose eyes are placed laterally (Clarke et al. 1976; Hughes 1977; Ramachandran et al. 1977; Pettigrew et al. 1984; Timney and Keil 1999; Martin 2009; Nityananda and Read 2017). A lesser frontalization of the eyes means a much larger area devoted to monocular cortical representation, as is the case of the cat compared to primates (Sanides and Hoffmann 1969; Fox and Blake 1971). Nonetheless, neurons sensitive to ocular disparity have been found in the sheep and goat (Clarke and Witteridge 1976; Clarke et al. 1976, 1979a, b). The 60° binocular field (overlap) of goats suggests that their high agility could rely on stereopsis (Howard and Rogers 1996), although no behavioral evidence seems to exist, unlike in the horse (Timney and Kiel 1992, 1999), in which binocular vision reaches 80° (Harman et al. 1999) (see Table 1).

The cost in terms of neuronal circuitry for stereopsis is substantial (Nityananda and Read 2017); therefore, the cortical architecture of mammals could be a reliable predictor for the existence of stereopsis, which is not easily demonstrated from eye features or behavioral experiments. Despite the lack of direct link between orbital plane and eye field (Hughes 1977), the gross segregation we made based on eye position (Fig. 31) did correlate quite strongly with the results obtained for the V1 cytoarchitecture morphometrics of the different species (Fig. 32). In particular, lateral-eyed specimens (cetaceans and amphibious species) had a notably lower density (Fig. 32, *p* ≤ 0,01 for all but L1) and larger neuron size (perimeter and area in Fig. 32). Wide field animals (all terrestrial, all ungulates) had significantly lower density compared to frontal-eyed animals (primates), but were relatively similar in terms of size. Interestingly, wide-field animals had much more slender neurons (indicated by Extent in Fig. 32) than both frontal-eyed and lateral-eyed ones, which would tend to suggest the prevalence of pyramidal cells, or alternatively the relative lack of round granule cells as in primates.

The proposed statistical approach is nonparametric and multivariate in nature, it allows for a flexible and hierarchical exploration of the results while providing a valid inference in a multiple testing paradigm. In spite of this, in this work, the phylogenetic relatedness of the data are not accounted for. The lack of interaction of the relative phylogenetic distance on the data may partially dilute the solidity of the results. Within the parametric framework, the recommended tool is the phylogenetic generalized least squares (Symonds et al. 2014). The proposed method can account for this information (Winkler et al. 2014) as well. In spite of this, collecting phylogenetic information may be demanding, costly and was beyond the scope of this work. We do not exclude to use these in future work to further corroborate our claims.

The eyes of marine Cetartiodactyls are invariably placed laterally, allowing panoramic (120–130° on each side) view but little to no binocularity, with independent eye, eyelid and pupil movement (Mass and Supin 2007). As per the low density of ganglion neurons in the retina (about 500–750 per mm2, Murayama et al. 1992; Mass and Supin 2007) compared to terrestrial Cetartiodactyls (Hebel 1976; Shinosaki et al. 2010), the optic nerve is distinctively composed of fewer larger fibers in cetaceans compared to terrestrial mammals and humans (Dawson et al. 1983; Dral 1983; Jonas et aI. 1990; Mass and Supin 1990, 1997). Fibers in the optic nerve completely decussate in the chiasma and run contralaterally in the bottlenose dolphin. Some doubts remain in other species such as the porpoise (*Phocoena phocoena*) or to a lesser degree in the minke whale (Morgane and Jacobs 1972). A narrow binocular field of view corresponding to the temporal opening of the opercular-shaped iris, and possible active accommodation were described in the bottlenose dolphin (Dral 1975). Additionally, bottlenose dolphins seem to visually recognize their surrounding environment similarly to primates (Tomonaga et al. 2014). Therefore, in selected delphinid species, the theoretical possibility of stereopsis could exist on these grounds but remains to be demonstrated. However, stereopsis must be excluded in species endowed with a large head and completely lateral eye, hence without binocular field, as the sperm whale and the fin whale.

It was clearly demonstrated that LGN connections are ipsilateral to at least V1, V2 and V3 in the cat and monkey (Wilson 1968). The few binocular cell studies have shown that in animals with almost total decussation, binocular cells have little in common with that of frontal-eyed species such as the cat or primates (Van Sluyters and Stewart 1974). On the other hand, Morgane and Jacobs (1972) noted a significant loss (to the point of apparent *disappearance*) of fibers at the chiasmatic level (up to 19% in the minke whale). These missing fibers might run to the hypothalamus and constitute a retino-hypothalamic connection, which is well known to exist in mammals and form a loop between the retinal ganglion cells, the suprachiasmatic nucleus and the intergeniculate leaflet of the thalamus (Pickard 1985). This connection alone is not sufficient to explain a 19% decrease, since the connection is made of collateral branches or retinofugal axons, which also reach the optic tract. The prevalence of retinothalamic projections compared to the incipient pretectal connection has also been compared to a “primate-like” pattern rather than that of carnivores. The latter was judged “puzzling” by Jacobs et al. (1975) given the strong pupillary light reflex noted by Dral (1972).

The canonical paradigm is that magnocellular (M), parvocellular (P) and koniocellular (K) ganglion cells provide separate routes through the optic nerve and the lateral geniculate nucleus, to reach separated sublayers in L4 and L3 of the visual cortex (Sincich and Horton 2005). However, L4 is all but evanescent in Cetartiodactyls and especially in cetaceans, with significant variations among species as portrayed in the present study. This poses the question of how these putative inputs reach the dedicated cortex. Since their retina seems to contain only L-opsins and rods (Jacobs 2009; Springer et al. 2016) implying colorblindness, does there remain separate pathways, which could be seen in the LGN and ending in different cortical layers? The retina comprises giant ganglion cells (Dawson 1973) and smaller ones, and different neuron sizes can have been reported in the LGN (Kruger 1959; Jacobs et al. 1975). Additionally, cetaceans could compensate their lack of cortical specialization by an increase in cortical surface (Morgane et al. 1990), and thereby explaining the myeloarchitectonic increase of L6, known to be the cortico-cortical connection site. Wiring strategies in developing vision and hearing follow different routes and targets (Rygvold et al. 2021; Sitko and Goodrich 2021) and may also rely on specific variation in the cortical column based on the presence and distribution of immature neurons in L2 and potential plasticity (La Rosa et al. 2020).

## Conclusions

Our data suggest that the lateral position of the eyes in Cetartiodactyls is accompanied by specific organization, structure, and complexity of the visual cortex. Overall, said characteristics include lesser lamination, diminished density, and general apparent simplification of the cortical column. Changes are not linear across species, but comparison between Cetartiodactyls and Perissodactyls indicate either a feature present in a common ancestor, or a substantial parallel evolution, contrarily to what observed in primates.

Cetaceans, usually considered altogether as a group, include species (Families?) that present several types of cortical lamination which may reflect differences in function. Activity patterns of the sensory systems seems to heavily influence relative brain and neuronal allocation (Karlen and Krubitzer 2009). Toothed whales rely on echolocation for orientation and hunting, an activity than may be independent from the support of sight, at least in the Cuvier’s beaked whale and in the sperm whale. Moreover, all cetaceans live in the aquatic environment, and inputs to V1 may reflect environmental conditions of fading luminance and contrast (Tang et al. 2021) due to light progressively absorbed with increasing depth of the water column.

## Data Availability

Data can be made available upon request to the authors.
